# Inorganic Dielectric Materials Coupling Micro‐/Nanoarchitectures for State‐of‐the‐Art Biomechanical‐to‐Electrical Energy Conversion Devices

**DOI:** 10.1002/adma.202419081

**Published:** 2025-05-03

**Authors:** Jia‐Han Zhang, Zhengtong Li, Zeng Liu, Mingxuan Li, Jiaxin Guo, Jinhua Du, Changkun Cai, Shaohui Zhang, Ningning Sun, Yong Li, Xingtao Xu, Xihong Hao, Yusuke Yamauchi

**Affiliations:** ^1^ School of Electronic Information Engineering Electronic‐Photonic Smart Sensing Device R&D Team Inner Mongolia Key Laboratory of Intelligent Communication and Sensing and Signal Processing Inner Mongolia University Hohhot 010021 China; ^2^ Key Laboratory of Hydrology Water Resources and Hydraulic Engineering Hohai University Nanjing 210098 China; ^3^ School of Chemistry and Chemical Engineering Inner Mongolia University of Science and Technology Baotou 014010 China; ^4^ Inner Mongolia Key Laboratory of Advanced Ceramic Materials and Devices, School of Materials Science and Engineering Inner Mongolia University of Science and Technology Baotou 014010 China; ^5^ National Engineering Research Center for Healthcare Devices & Guangdong Provincial Key Laboratory of Medical Electronic Instruments and Materials Institute of Biological and Medical Engineering Guangdong Academy of Sciences Guangzhou 510316 China; ^6^ China Marine Science and Technology College Zhejiang Ocean University Zhoushan 316022 China; ^7^ Australian Institute for Bioengineering and Nanotechnology (AIBN) The University of Queensland St. Lucia Brisbane Queensland 4072 Australia; ^8^ Department of Materials Process Engineering Graduate School of Engineering Nagoya University Nagoya 464–8603 Japan

**Keywords:** biomechanical‐to‐electrical energy conversion, inorganic dielectric materials, micro‐/nanoarchitectures

## Abstract

Biomechanical‐to‐electrical energy conversion technology rapidly developed with the emergence of nanogenerators (NGs) in 2006, which proves promising in distributed energy management for the Internet of Things, self‐powered sensing, and human–computer interaction. Recently, researchers have increasingly integrated inorganic dielectric materials (IDMs) and micro‐/nanoarchitectures into various types of NGs (i.e., triboelectric, piezoelectric, and flexoelectric NGs). This strategy significantly enhances the electrical performance, enabling near‐theoretical energy harvesting capability and precise multiple physiological information detection. However, because micro‐/nanoarchitectured IDMs function differently in each type of NG, numerous studies have focused on a single NG type and lack a unified perspective on their role across all types of biomechanical energy NGs. In this review, from an overall theoretical root of NGs, the performance enhancement mechanisms and effects of designs of IDMs coupling micro‐/nanoarchitectures of various kinds of biomechanical energy NGs are systematically summarized. Next, advanced applications in human energy scavenging and physiological signal sensing are delved into. Finally, challenges and rational guidelines for designing future devices are discussed. This work provides researchers with in‐depth insight into the development of high‐performance personalized high‐entropy power supplies and sensor networks via biomechanical‐to‐electrical energy conversion technologies based on IDMs coupling micro‐/nanoarchitectures.

## Graphical abstract

Biomechanical‐to‐electrical energy conversion devices are uniquely suited for self‐driven physiological information monitoring and powering human–computer interaction systems. These devices based on micro‐/nanoarchitectured inorganic dielectric materials (MNIDMs) have shown ultrahigh electromechanical performance and thus great potential for practical deployment. This review constructs a nexus among MNIDMs and all kinds of biomechanical‐to‐electrical energy conversion nanogenerators in terms of material development.

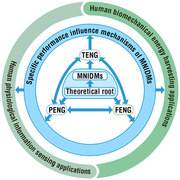

## 1 Introduction

**Figure 1:**
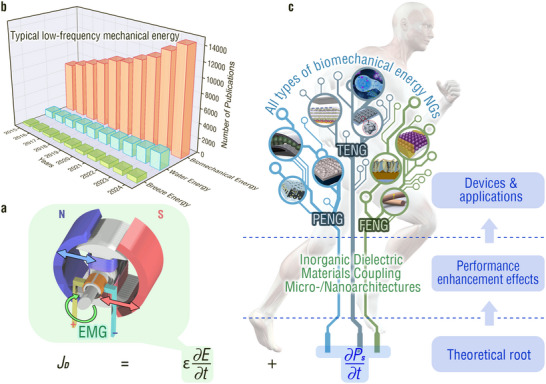
a) Schematic diagram of an electromagnetic generator. b) Number of papers regarding three types of typical low‐frequency mechanical energies over the past decade (collected from ISI Web of Science). c) Micro‐/nanoarchitecture of biomechanical‐to‐electrical energy conversion devices based on inorganic dielectric materials. Equation in the bottom corner is the Maxwell's displacement current equation. The first term *ɛ*∂*E*/∂*t* is responsible for the electromagnetic field theory; and the second term ∂*P*_s_/∂*t* is the foundation of nanogenerators. Reproduced with permission.^[^ ^21^ ^]^ Copyright 2015, Elsevier. Reproduced with permission.^[^ ^22^ ^]^ Copyright 2018, American Chemical Society. Reproduced with permission.^[^ ^23^ ^]^ Copyright 2014, American Chemical Society. Reproduced with permission.^[^ ^24^ ^]^ Copyright 2020, American Chemical Society. Reproduced with permission.^[^ ^25^ ^]^ Copyright 2022, American Chemical Society. Reproduced with permission.^[^ ^26^ ^]^ Copyright 2018, American Chemical Society. Reproduced with permission.^[^ ^27^ ^]^ Copyright 2022, Elsevier. Reproduced with permission.^[^ ^28^ ^]^ Copyright 2018, Elsevier. Copyright 2022, Elsevier. Reproduced with permission.^[^ ^29^ ^]^ Copyright 2019, Elsevier.

The rapid development of the Internet of Things (IoT) has revolutionized the energy structure of humans.^[^ ^1^ ^]^ Traditional high‐frequency mechanical energies are converted into electrical energy via immovable giant electromagnetic generators (EMGs, **Figure** **1**a), which are not sufficient to meet the flexible and distributed energy demands of the IoT.^[^ ^2^ ^]^ In contrast, low‐frequency mechanical energies (usually below 10 Hz), such as breezes, water waves, and human motion, are ubiquitous in the environment and can render flexible and distributed energy supplies to the IoT.^[^ ^3^ ^]^ Among them, human biomechanical energy stands out as an optimal candidate for powering microdevices in future human–machine interaction networks,^[^ ^4^ ^]^ and has attracted growing attention over the past decade (Figure 1b).

A feasible biomechanical‐to‐electrical energy conversion device must be flexible, light, small, and biocompatible to meet the requirements of wearability or implantability with minimum interference with natural human movement and manipulation.^[^ ^5^ ^]^ In 2006, Wang et al. developed piezoelectric nanogenerators (PENGs), which revealed the possibility of converting biomechanical energy into electrical energy at the nanoscale.^[^ ^6^ ^]^ Following this, triboelectric nanogenerators (TENGs) and flexoelectric nanogenerators (FENGs) were developed to expand the range of materials and mechanisms for biomechanical energy conversion.^[^ ^7^ ^]^ Their flexible and small structures, sensitive responses, and high energy conversion efficiencies at low frequencies make them ideal for various applications, especially in biomechanical energy harvesting and self‐powered sensing.^[^ ^8^ ^]^

Electrical output is a key indicator of the performance of TENGs, PENGs, and FENGs.^[^ ^9^ ^]^ According to their theoretical root of Maxwell's displacement current (associated with the polarization of electrostatic surface charges as a result of mechanical triggering,^[^ ^10^ ^]^ Figure 1c), polarization dominates the electrical output performance.^[^ ^11^ ^]^ Over the years, to enhance the electrical output performance, research has increasingly focused on integrating these NGs with inorganic dielectric materials (IDMs), such as lead zirconate titanate (PZT), barium titanate (BT), and zinc oxide (ZnO), owing to their inherent excellent polarization response.^[^ ^12^ ^]^ By further coupling IDMs with functional micro‐/nanostructures, NGs possess superior polarization regulation capabilities.^[^ ^13^ ^]^ For example, the large contact area arising from micro‐/nanoarchitectured IDM (MNIDM) tribo‐materials can result in a high surface polarization.^[^ ^14^ ^]^ Piezoelectric ceramics with 3D interconnected microskeleton structures have outstanding load‐transfer abilities, and therefore better piezoelectric polarization responses.^[^ ^15^ ^]^ Micro‐/nanoarchitectures are also important for creating an essential mechanical strain gradient so that inorganic flexoelectric materials can fully exhibit flexoelectric polarization.^[^ ^16^ ^]^ Moreover, MNIDMs possess superb wearability in biomechanical‐to‐electrical energy conversion. The fabrication of ultrathin,^[^ ^9b^ ^]^ granular,^[^ ^17^ ^]^ strip‐like,^[^ ^18^ ^]^ or wire‐like^[^ ^6^ ^]^ inorganic micro‐/nanodielectric materials provides an effective strategy for realizing device flexibility, and fibrous IDM‐based devices achieve good breathability and comfort.^[^ ^19^ ^]^ Therefore, the strategy of coupling IDMs with micro‐/nanoarchitectures has great potential for producing next‐generation high‐performance biomechanical‐to‐electrical energy conversion devices.

Although many reviews have discussed MNIDM‐based NGs for biomechanical‐to‐electrical energy conversion, they focused only on the effects of IDMs on single‐type NGs.^[^ ^7^, ^20^ ^]^ A comprehensive analysis of how IDMs and micro‐/nanoarchitectures synergistically enhance the performance of all types of biomechanical energy NGs (including TENGs, PENGs, and FENGs) is still lacking. Meanwhile, no previous report elucidates the intrinsic theoretical connections and structure–performance relationship differences of the various MNIDM‐based biomechanical energy NGs. TENGs, PENGs, and FENGs have the same theoretical root yet differ in their operation modes and responses to external stimuli.^[^ ^21^, ^22^, ^23^, ^24^, ^25^, ^26^, ^27^, ^28^, ^29^ ^]^ A comprehensive analysis of these NGs can bridge knowledge gaps and provide deeper insights into the role of IDMs coupling micro‐/nanoarchitectures in various biomechanical‐to‐electrical energy conversion technologies. It will guide the development of the most suitable devices for specific application scenarios and multi‐effect coupled devices for efficient human biomechanical energy scavenging and effective self‐powered physiological information sensing. Therefore, it is necessary to provide a comprehensive overview of various biomechanical‐to‐electrical energy conversion NGs based on MNIDMs.

In this Review, we first discuss the commonalities among TENGs, PENGs, and FENGs in terms of first principles, abstracted capacitor models, and output characteristics. We then analyze the diversity in their operation modes and output electrical performance determinants. Subsequently, corresponding to the performance determinants, the unique progressiveness of IDMs coupling micro‐/nanoarchitectures for these NGs is highlighted with a focus on various performance enhancement effects. In addition, we summarize the promising applications of MNIDM‐based TENGs, PENGs, and FENGs in human biomechanical energy harvesting and physiological information detection. Finally, we analyze the remaining challenges, opportunities, and future directions of MNIDM‐based devices for human biomechanical‐to‐electrical energy conversion.

## 2 Nanogenerators for Mechanical‐to‐Electrical Energy Conversion

**Figure 2:**
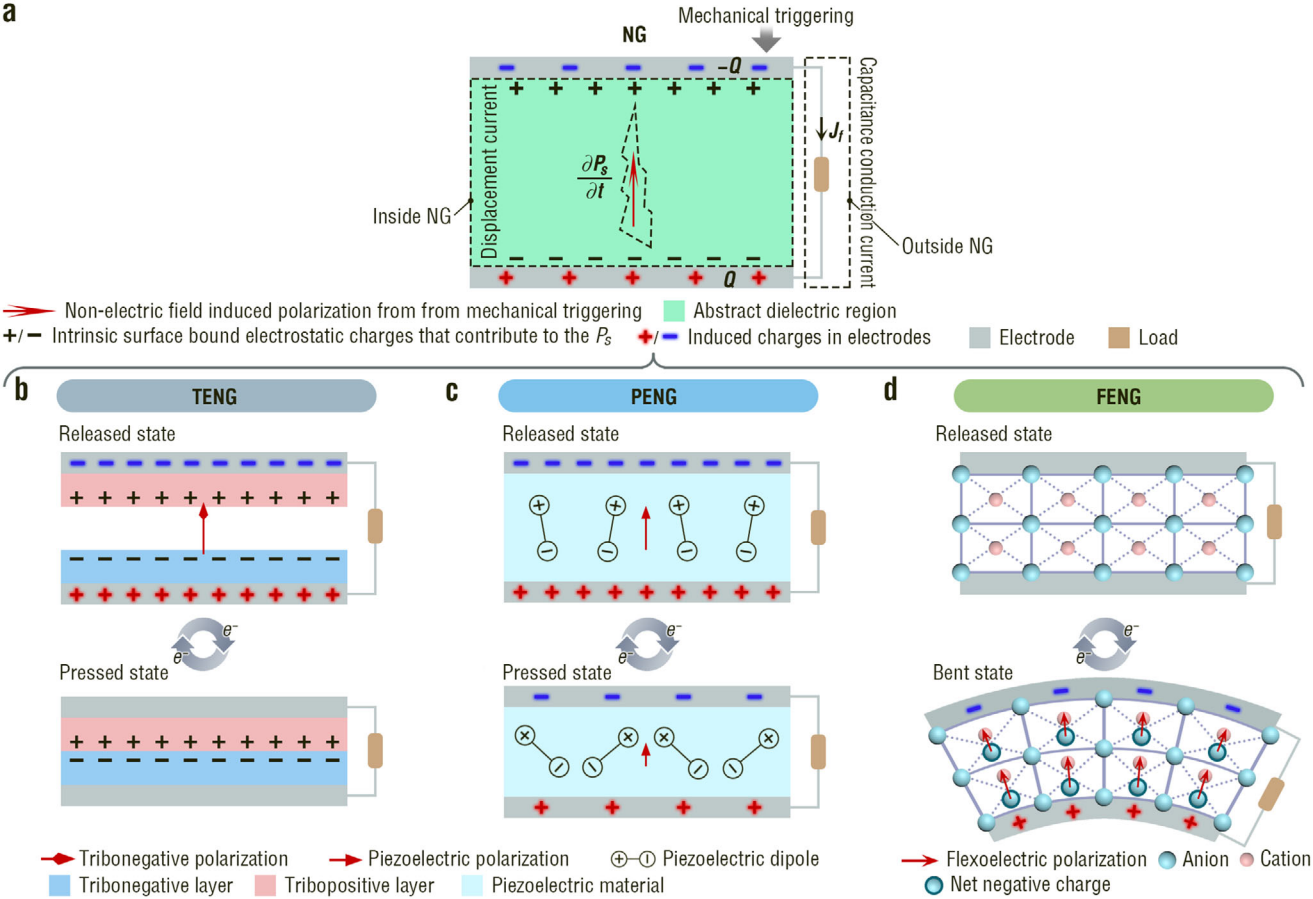
First principle and working mechanisms of three types of nanogenerators. a) First principle of nanogenerators. ∂*P*_s_/∂*t* is displacement current density. *J*_f_ is free electric current density. b,c) Working mechanisms of (b) Triboelectric nanogenerators, (c) piezoelectric nanogenerators, and (d) flexoelectric nanogenerators.

In general, NGs, including TENGs, PENGs, and FENGs, consist of electrodes and dielectric materials that can effectively scavenge biomechanical energy.^[^ ^21^, ^22^, ^23^, ^24^, ^25^, ^26^, ^27^, ^28^, ^29^, ^30^ ^]^ The first principle of TENGs, PENGs, and FENGs is Maxwell's displacement current, which serves as the driving force for converting mechanical energy into electrical energy/signals. Compared to other mechanical‐to‐electrical energy conversion devices that rely on Maxwell's displacement currents, the unique feature of NGs is their non‐electric‐field‐induced polarization (*P*_s_), stemming from intrinsic surface‐bound electrostatic charges due to media movement. To highlight this feature, Wang added the term ∂*P*_s_/∂*t* to Maxwell's equations:^[^ ^30^ ^]^

(1) $$J_{D}=\frac{\partial D}{\partial t}=\varepsilon_{0}\frac{\partial E}{\partial t}+\frac{\partial P_{s}}{\partial t}$$

where *J_D_* is the total displacement current density, *D* is the electric displacement vector (i.e., displacement field), *E* is the electric field, *t* is the time, and *ɛ*_0_ is the dielectric constant in a vacuum. As shown in **Figure** **2**a, the TENG, PENG, and FENG were abstracted into a capacitor model. The displacement current inside the NG is the result of nonelectric‐field‐induced polarization and media movement. Outside the NG, a capacitive conduction current is observed, which is an external manifestation of the displacement current. No matter the TENG, PENG, and FENG, the potential drop in an external load (*φ*) and the output power (*p*) meet the following two equations:^[^ ^10^ ^]^

(2) $$\varphi =\frac{\partial Q}{\partial t}R$$

(3) $$p={\left(\frac{\partial Q}{\partial t}\right)}^{2}R$$

where *Q* is the total free charge on the electrode (i.e., the amount of transferred charge) and *R* is the resistance of the load.

The abstracted capacitor model of the NG (Figure 2a) is derived from a TENG, PENG, and FENG by replacing the abstracted dielectric region with different dielectric materials. i) A pair of tribolayers with oppositely bound charges resulting from contact electrification and a variable gap dependent on the mechanical movement are responsible for the displacement current of a TENG (Figure 2b). ii) Uniform strain‐induced piezoelectric polarization in the dielectric layer contributes to the displacement current of a PENG (Figure 2c). iii) The flexoelectric displacement current in a centrosymmetric crystal is reflected by the polarization under an inhomogeneous strain (Figure 2d). These three types of NGs possess distinct structures and, therefore, different operating modes. Their operation modes and corresponding output characteristics are discussed in Sections 2.1–2.3.

### 2.1 Operation Mode and Output Characteristics of TENGs

A TENG is based on the coupling effect of contact electrification and electrostatic induction.^[^ ^31^ ^]^ According to the type of device structure, they can operate in four modes, viz., vertical contact–separation, lateral‐sliding, single‐electrode, and free‐standing mode.^[^ ^32^ ^]^ We choose the most common mode, the vertical contact–separation mode with two different dielectric tribolayers, as a simple case. As shown in Figure 2b, an equal amount of opposite charges bound on a pair of tribolayer surfaces arise from the charge transfer between the two tribolayers in contact with each other owing to the triboelectrification effect.^[^ ^33^ ^]^ These bound surface electrostatic charges usually reach saturation after dozens of contact–separation cycles of the two tribolayers.^[^ ^34^ ^]^ In the released state, the two tribolayers are separated and the gap between them reached the maximum allowable distance. Owing to the electrostatic induction effect, induced free charges appear in the two electrodes to screen the triboelectric field and achieve electrical equilibrium.^[^ ^35^ ^]^ In the pressed state, two tribolayers with equal amounts of positive and negative charges are in contact. There is no electrical potential between the two tribolayers, and therefore, no net free charge is present in the electrodes.^[^ ^36^ ^]^ When a TENG moves cyclically between the released and pressed states under a mechanical force, the electrons in the external circuit must continuously flow to maintain electrical equilibrium. Consequently, the classic TENG outputs alternating current (AC) signals.^[^ ^37^ ^]^

The fundamental electrical characteristics of a contact‐separation mode TENG can be expressed as follows:^[^ ^38^ ^]^

(4) $$V=-\frac{Q}{S\varepsilon_{0}}\left(h_{0}+x\left(t\right)\right)+\frac{\sigma x(t)}{\varepsilon_{0}}$$

(5) $$h_{0}=\sum_{i=1}^{n}\frac{h_{i}}{\varepsilon_{\mathrm{ri}}}$$

where *V* is the voltage between the two electrodes, *S* is the area of electrodes, *x* is the distance between the two triboelectric charged layers, *σ* is the tribocharge surface density, *h*_0_ is the effective thickness constant, and *n* is the number of the dielectric tribolayers in a contact–separation‐mode TENG: *n* = 1 and 2 for a TENG with one and two dielectric tribolayers, respectively, *h_i_* is the thickness of the corresponding dielectric tribolayer, and *ɛ*_r_ *_i_* is the permittivity (also known as relative dielectric constant) of the corresponding dielectric tribolayer. Equations (4) and (5) show the *V*–*Q*–*x* relationship of a TENG. Accordingly, the open‐circuit voltage (*V*_OC_), short‐circuit current (*I*_SC_), and amount of transferred charge under short‐circuit conditions (*Q*_SC_) are deduced as follows:^[^ ^11c^ ^]^

(6) $$V_{\mathrm{OC}}=\frac{\sigma \;x(t)}{\varepsilon_{0}}$$

(7) $$I_{\mathrm{SC}}=\frac{S\;\sigma \;h_{0\;}v(t)}{{(h_{0}+x(t))}^{2}}$$

(8) $$Q_{\mathrm{SC}}=\frac{S\;\sigma \;x(t)}{h_{0}+x(t)}$$

where *v*(*t*) is the speed of movement of the tribolayer. Furthermore, the transferred charge amount *Q* can be solved analytically as follows:^[^ ^38^ ^]^

(9) $$\begin{array}{c} Q(t)=\;\sigma S\;-\;\sigma S\;\;\exp\;[-\frac{1}{RS\varepsilon_{0}}(h_{0}t\;+\int_{0}^{\;t}x(t)dt)]\\ \;\;-\;\frac{\sigma h_{0}}{R\varepsilon_{0}}\;\exp\;[-\frac{1}{RS\varepsilon_{0}}(h_{0}t\;+\int_{0}^{t}x(t)dt)]\\ \;\;\times \;\int_{0}^{t}\;\exp\;[\frac{1}{RS\varepsilon_{0}}(h_{0}t\;+\int_{0}^{t}x(t)dt)]dt \end{array}$$

Moreover, the analytical solutions of the output current *I* and output voltage *V* can be derived as follows:^[^ ^39^ ^]^

(10) $$I\left(t\right)=\frac{\mathrm{dQ}}{\mathrm{dt}}$$

(11) V(t)=RI(t)

They concluded that the transferred charge, current, and voltage of a TENG are proportional to the tribocharge surface density.

### 2.2 Operation Mode and Output Characteristics of PENGs

PENGs are based on the presence of piezoelectric dipoles, typically owing to the non‐centrosymmetric positive and negative charges within unit cell planes.^[^ ^40^ ^]^ Of the 32 crystallographic point groups that rely on the geometry and symmetry of the unit cell, 21 are non‐centrosymmetric, all but one (owing to other symmetry elements) of which present piezoelectricity.^[^ ^41^ ^]^ Some crystals with wurtzite structures, such as ZnO and gallium nitride (GaN), are typical piezoelectric materials used in PENGs.^[^ ^42^ ^]^ These piezoelectrics are nonferroelectric materials. In the released state, no piezoelectric polarization is observed for this type of PENG. Once an external force acts on the PENG, a relative displacement occurs between the cations and anions in the unit cells, leading to a piezoelectric polarization potential. In an external circuit, electrons are driven by a polarization potential to screen the piezoelectric field until electrical equilibrium (i.e., the pressed state) is achieved.^[^ ^43^ ^]^ When the PENG operates between the released and pressed states, AC signals are observed in the external circuit.

Moreover, 10 of non‐centrosymmetric crystallographic point groups have nonvanishing electric dipole moments in their unit cells, which show spontaneous polarization without mechanical stress and are termed ferroelectrics. Many of the most well‐known ferroelectrics, such as BT and PZT, possess perovskite crystal structures. PENGs based on these ferroelectrics exhibit different operating modes. In general, because the piezoelectric dipoles in primary ferroelectrics are randomly oriented, there is no net macroscopic polarization.^[^ ^35^ ^]^ The primary ferroelectrics undergo an electrical poling process that orients the crystal domains and aligns the dipoles in the same direction to exhibit piezoelectricity.^[^ ^40^ ^]^ Therefore, the driving force of the piezoelectric outputs for this type of PENG is the change in the ferroelectric polarization between the released and pressed states (Figure 2c).^[^ ^44^ ^]^ Recently, Park et al. achieved piezoelectricity in centrosymmetric Gd‐doped CeO_2−x_ (CGO) by applying an external electric field that breaks inversion symmetry.^[^ ^45^ ^]^ CGO has a record‐breaking piezoelectric coefficient of 200 000 pm V^−1^,^[^ ^46^ ^]^ making it a highly promising candidate for biomechanical‐to‐electrical energy conversion.

The relationship among basic electrical performances of a PENG is usually expressed as follows:^[^ ^13a^ ^]^

(12) $$Q_{i}=d_{\mathrm{ij}}\;S\;T_{j}$$

(13) $$d_{\mathrm{ij}}=\frac{P_{i}}{T_{j}}$$

where *Q*_i_ is the effective surface charge induced by the piezoelectric polarization along the i direction when mechanical stress *T*_j_ is applied stress along the j direction, *d*_ij_ is the piezoelectric coefficient, and *P*_i_ is the piezoelectric polarization along the i direction. There are three common energy‐conversion modes for PENGs. In the 33‐mode (i.e., i = 1, j = 1), the directions of the applied stress and generated voltage are the same. In the 31‐mode, the directions of the applied stress and generated voltage are perpendicular to each other. In the 15‐mode, the piezoelectric material is subjected to shear stress. The *V*_OC_ of a 33‐mode PENG can be deduced according to Equations (14) and (15), which show the relationship between charge, capacitance, and voltage.^[^ ^40^ ^]^

(14) $$Q_{i}=CV_{e}$$

(15) $$C=\frac{S\;\varepsilon_{r\;}\varepsilon_{0}}{h_{p}}$$

(16) $$V_{\mathrm{OC}}=\frac{d_{33}\;h_{p\;}T}{\varepsilon_{0}\;\varepsilon_{r}}$$

where *C* is the capacitance of the device, *V*_e_ is the voltage between the opposite electrodes of the capacitor, *ɛ*_r_ is the permittivity of the piezoelectric material, *h*_p_ is the thickness of the piezoelectric material, and *T* is the applied stress. The energy stored in the piezoelectric material (*W*_p_) that results from applied stress *T* can be approximate as:^[^ ^5^ ^]^

(17) $$W_{p}=\frac{1}{2}C\;V_{e}=\frac{d_{33}^{2}\;T^{2}\;S\;h_{p}}{2\;\varepsilon_{0}\varepsilon_{r}}$$

In addition, the *I*_SC_ can be written as:^[^ ^40^ ^]^

(18) $$I_{\mathrm{SC}}=\frac{dQ_{i}}{dt}=d_{33}S\frac{dT}{dt}$$

It has been concluded that the current, voltage, and stored energy of the PENG are positively associated with the piezoelectric coefficient.

### 2.3 Operation Mode and Output Characteristics of FENGs

A FENG is based on flexoelectric polarization induced by a strain gradient or nonuniform strain field that locally breaks the inversion symmetry of the material.^[^ ^13b^ ^]^ The flexoelectric effect is size‐dependent and becomes more significant at the nanoscale.^[^ ^47^ ^]^ Unlike piezoelectrics, flexoelectrics do not require a non‐centrosymmetric crystal structure.^[^ ^48^ ^]^ Therefore, a wide range of micro‐/nanomaterials exhibit flexoelectricity.^[^ ^9c^ ^]^ Depending on the type of mechanical structure that can cause nonuniform strain, there are three basic operation modes: transverse, axial, and shear mode.^[^ ^7b^ ^]^ Considering the simplest transverse mode (Figure 2d) as an example, when a slab FENG is in the released state, the centers of the positive and negative charges are coincident, the flexoelectric polarization is zero and no net charge is present in the electrodes. Once the slab FENG is bent, the upper part of the slab is subjected to tensile strain, and the lower part experiences compressive strain, building a strain gradient and resulting in the separation of the centers of positive and negative charges.^[^ ^16^ ^]^ Flexoelectric polarization occurs and induces net charges in the electrodes. When a FENG operates between these two states under a mechanical force, AC signals are detected in the external circuit.^[^ ^28^ ^]^

The relationship among basic electrical performances of a FENG is usually expressed as follows:^[^ ^9^, ^49^ ^]^

(19) $$P_{l}=\mu_{\mathrm{ijkl}}\frac{\partial T_{\mathrm{ij}}}{\partial x_{k}}$$

(20) $$Q_{f}=\int \mu_{\mathrm{ijkl}}{\frac{\partial T_{\mathrm{ij}}}{\partial x_{k}}}_{l}dS$$

where *P*_l_ is the induced flexoelectric polarization, *µ*_ijkl_ is the flexoelectric coefficient, *T*_ij_ is the elastic strain, *x*_k_ is the position coordinate, and *Q*_f_ is the flexoelectrically induced charge on the electrode. The flexoelectric coefficient is an important indicator of the output performance of FENGs.^[^ ^11^, ^13^, ^50^ ^]^

## 3 Inorganic Dielectric Materials Coupling Micro‐/Nanoarchitectures for NGs

The term IDM refers to an inorganic material that can be polarized under an external electric field.^[^ ^51^ ^]^ This category includes inorganic insulators (e.g., PZT and BT)^[^ ^52^ ^]^ and inorganic semiconductors (e.g., ZnO and TiO_2_).^[^ ^16^, ^53^ ^]^ Compared to most organic dielectric materials,^[^ ^54^ ^]^ IDMs have special crystal structures,^[^ ^4^, ^13^ ^]^ which provide not only superior polarization regulation^[^ ^24^ ^]^ but also outstanding environmental tolerance at high temperatures,^[^ ^55^ ^]^ under intense radiation,^[^ ^56^ ^]^ and against aging.^[^ ^9^, ^20^ ^]^ The development and enhancement of these special properties of IDMs usually rely on specific micro‐/nanoarchitectures owing to their unique mechanical and electrical adjustment capabilities.^[^ ^19^, ^29^, ^57^ ^]^ Micro‐/nanoarchitectures enhance the dielectric properties,^[^ ^12f^ ^]^ flexibility, and robustness of IDMs.^[^ ^12^, ^18^, ^58^ ^]^ Dielectric polarization directly determines the tribocharge surface density, piezoelectric coefficient, and flexoelectric coefficient of TENGs, PENGs, and FENGs, as described in Equations ((4), (5), (6), (7), (8), (9), (10), (11), (12), (13), (14), (15), (16), (17), (18), (19), (20)). Flexibility and robustness are also critical for wearable applications. Therefore, the construction of MNIDMs is regarded as an ideal strategy for obtaining NGs with outstanding biomechanical energy utilization capability and wearability. In the following sections, the various positive effects of MNIDMs on TENGs, PENGs, and FENGs are reviewed. **Table** **1** summarizes the specific improvements in the device output achieved through various strategies based on MNIDMs, and Table S1 (Supporting Information) outlines the material compatibilities and scalabilities of these strategies.

**Table 1:** Comparison of the effects of various performance optimization strategies for MNIDM‐based NGs.

NG types	Strategies	Materials	Optimized performances (efficiency, voltage, current, transferred charge, power, etc.)	Original performances	Refs.
TENG	Permittivity enhancement	ST particles, porous PDMS matrix	338 V, 9.06 µA cm^−2^, 19 nC cm^−2^, 647 µW cm^−2^	257 V, 6.6 µA cm^−2^, 13.2 nC cm^−2^	[59]
		BT nanoparticle‐PDMS‐PVDF composite nanofibers	1020 V, 1.32 µA cm^−2^, 5.8 nC cm^−2^, 220 µW cm^−2^	∼780 V, 0.682 µA cm^−2^, 3.7 nC cm^−2^	[60]
		Ti_0.87_O_2_ nanosheets, PVDF matrix	52.8 V, 5.69 µA cm^−2^, 92.5 µW cm^−2^	22 V, 0.73 µA cm^−2^, 1.85 µW cm^−2^	[61]
		BT particles, bacteria cellulose nanofibers	181 V, 2.06 µA cm^−2^, 7.52 nC cm^−2^, 48 µW cm^−2^	121 V, 0.98 µA cm^−2^, 3.44 nC cm^−2^	[62]
		PZT layer, glass fibers	1640 V, 5.905 µA cm^−2^, 10.29 nC cm^−2^, 1080 µW cm^−2^	540 V, 1.33 µA cm^−2^, 3.05 nC cm^−2^	[8b]
		Multilayer BT nanoparticle‐PVDF‐TrFE composite film	44 V, 1.77 µA cm^−2^, 29.4 µW cm^−2^	35.4 V, 1.2 µA cm^−2^	[24]
	Interface contact optimization	Yarn based on ZnO nanowalls and ZnO nanoparticles	69.5 V, 9.05 µA cm^−2^, 629 µW cm^−2^	13.1 V, 1.05 µA cm^−2^, 13.7 µW cm^−2^	[64]
	Electron trapping effect	MoSe_2_‐polyimide layer	32.82%, 1200 µA	300 µA	[120]
		TiO_x_ nanofilm, PDMS	272 V, 1.01 µA cm^−2^	50 V, 0.222 µA cm^−2^	[72]
		Monolayer MoS_2_, polyimide	400 V, 3.93 µA cm^−2^, 53.3 nC cm^−2^	30 V, 1.6 µA cm^−2^, 13.3 nC cm^−2^	[71b]
	Piezo/ferroelectric coupling	ZnO nanowire arrays, PVDF	34.56%, 40 V	24.35%, 16.7 V	[121]
		ZnO nanoflower, PDMS matrix	39.87 V, 0.258 µA cm^−2^	22.05 V, 0.173 µA cm^−2^	[26]
		PZT particles, PVDF‐TrFE matrix	330 V, 33.3 µA cm^−2^, 11.7 nC cm^−2^, 711 µW cm^−2^	28.4 V, 0.96 nC cm^−2^,	[74]
	Large polarization difference effect	Pb_0.94_La_0.04_Zr_0.98_Ti_0.02_O_3_ film, PVDF	∼43%, 456 V, 11.6 µA cm^−2^, 13.5 nC cm^−2^	217 V, 5.52 µA cm^−2^, 6.45 nC cm^−2^	[9b]
	Electric displacement modulation	PZT micro‐skeleton, PDMS matrix	∼45%, 394 V, 7.68 µA cm^−2^, 20.3 nC cm^−2^, 690 µW cm^−2^ 2.76 µJ cm^−2^	7.14 nC cm^−2^, 0.836 µJ cm^−2^	[76]
PENG	Permittivity optimization	Na_0.47_K_0.47_Li_0.06_NbO_3_ microcubes, PDMS matrix	11.12%, 48 V, 0.43 µA cm^−2^	N.A.	[17]
		KNLN microparticles, copper nanorods, PDMS matrix	12 V, 0.13 µA cm^−2^	3.4 V, 0.022 µA cm^−2^	[90a]
		Bi_2_WO_6_ nanoparticles, PDMS matrix	23.18%, 15 V	10 V	[122]
	Constructing local heterogeneity	NPR‐NNO	1098 pm V^−1^	22 pm V^−1^	[93]
	Inserting symmetry‐breaking defects	CGO thin film	200 000 pm V^−1^	∼0 pm V^−1^	[45]
	Boosting load‐transfer efficiency	3D interconnected PZT foam, PDMS matrix	85 V, 40 nA	7 V, 0.4 nA	[15a]
		ZnO nanoparticle‐PVDF fibers	91%, 26.7 µW cm^−2^, piezoelectric polarization 0.975 nC cm^−2^	Piezoelectric polarization 0.45 nC cm^−2^	[123]
	Piezoelectric & semiconducting property coupling	ZnO nanowire arrays	17%–30%, 7 mV for a single nanowire	N.A.	[6]
	Weakening screening effect	ZnO nanowire arrays	58 V, 134 µA cm^−2^	37 V, 12 µA cm^−2^	[105]
		ZnO nanowire arrays	0.284 V, 2.65 nA cm^−2^	0.0071 V, 0.415 nA cm^−2^	[53a]
	Poling effect enhancement	BT nanofibers, PDMS matrix	2.67 V, 0.17 µA cm^−2^, 0.12 µW cm^−2^	0.56 V, 0.026 µA cm^−2^, 0.0039 µW cm^−2^	[19]
	Electret/flexoelectric coupling	BT nanoparticles, porous PVDF matrix	25.66 V, 677.45 nA	1.70 V, ∼50 nA	^[^ ^107^ ^]^
		Buckled PZT ribbons	130 pm V^−1^	75 pm V^−1^	[108]
	Constructing multiple boundary interfaces	Sm‐PMN‐PT nanowires, PVDF matrix	169 nC cm^−2^, 290 µA cm^−2^	N.A.	[87h]
FENG	Autologous nonuniform structural design	Paraelectric BST micropyramid arrays	41 pC N^−1^ (pyramid height 50 µm)	19 pC N^−1^ (pyramid height 100 µm)	[114]
		BZT‐BCT microfiber clusters	9 V	N.A.	[28]
	Allosome nonuniform structural design	IDM film on a striated wrinkled PDMS substrate	Flexoelectric polarization 100 nC cm^−2^ for 20 nm‐thick ST film; 140 µC cm^−2^ for 10 nm‐thick BST film	Flexoelectric polarization 110 µC cm^−2^ for 20 nm‐thick BST film	[115]

### 3.1 TENGs Based on MNIDMs

Based on the discussion in Section 2.1, tribocharge density dominates the electrical performance of TENGs. Multiple effects of MNIDMs can elevate the charge density of the tribolayers. These typical effects include i) permittivity enhancement, ii) boosting of triboelectric polarity, iii) interface contact optimization, iv) electron trapping effect, v) dual‐effect coupling, vi) large polarization difference effect, and vii) electric displacement modulation.^[^ ^8^, ^9^, ^24^, ^26^, ^35^, ^52^ ^b,^ ^59^, ^60^, ^61^, ^62^, ^63^, ^64^, ^65^, ^66^, ^67^, ^68^, ^69^, ^70^, ^71^, ^72^, ^73^, ^74^, ^75^, ^76^ ^]^

#### 3.1.1 Permittivity Enhancement

**Figure 3:**
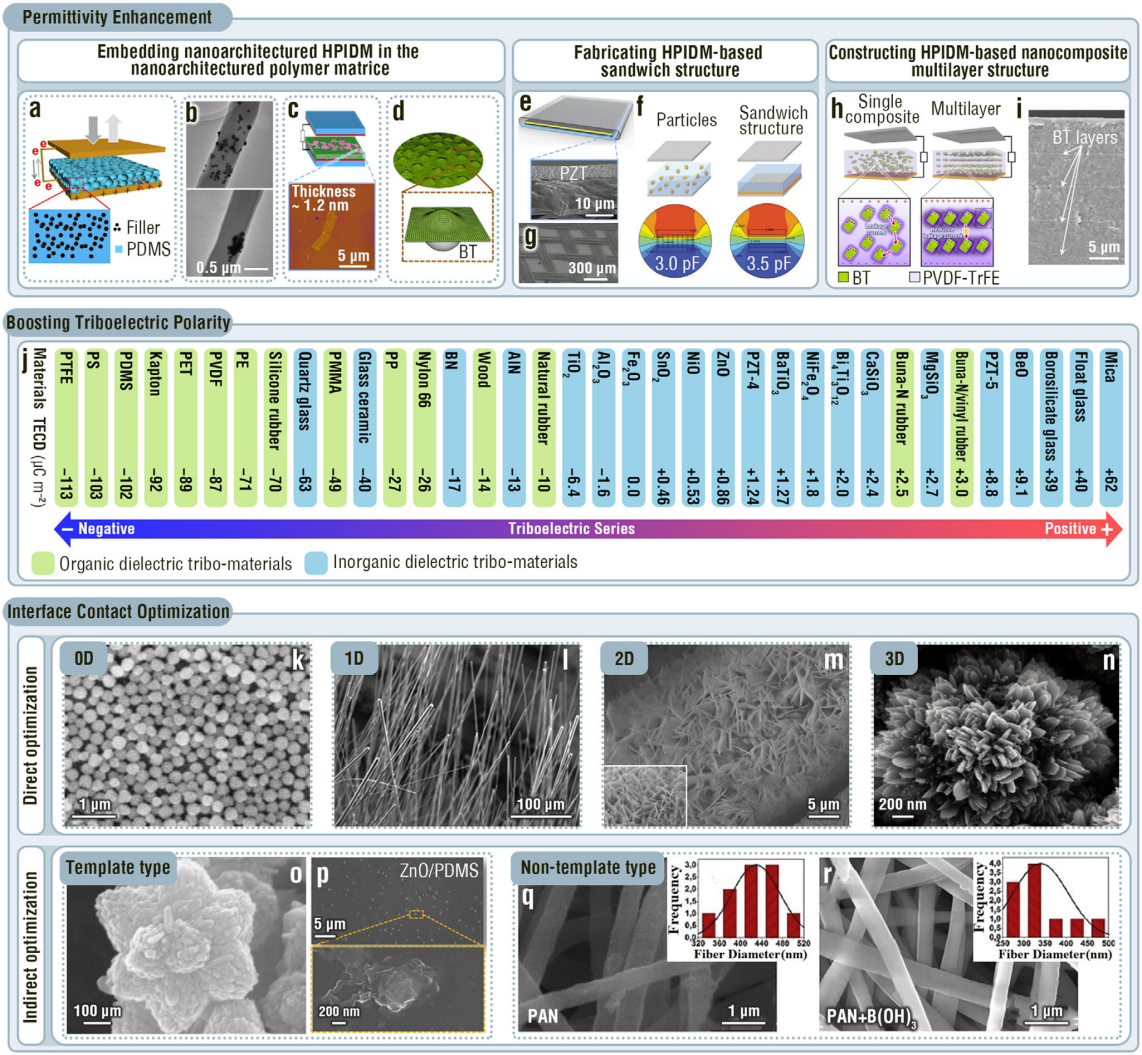
MNIDM‐based TENGs with improved permittivity, triboelectric polarity, and effective contact area. a–i) Permittivity enhancement effect of various MNIDMs on TENG outputs. a) Schematic diagram of the TENG based on the microporous IDM‐PDMS composite tribolayer. Reproduced with permission.^[^ ^59^ ^]^ Copyright 2016, American Chemical Society. b) Transmission electron microscope (TEM) images of electrospun nanofibers with uniformly distributed and agglomerated BT nanoparticles, respectively. Reproduced with permission.^[^ ^60^ ^]^ Copyright 2020, Elsevier. c) Schematic diagram of the TENG based on monolayer Ti_0.87_O_2_ sheet‐PVDF composite tribolayer and atomic force microscopy (AFM) image of a Ti_0.87_O_2_ sheet. Reproduced with permission.^[^ ^61^ ^]^ Copyright 2018, Elsevier. d) Schematic diagram of the bacteria cellulose‐BT composite film. Reproduced with permission.^[^ ^62^ ^]^ Copyright 2019, Elsevier. e) Structure scheme of the TENG with a PZT layer‐based sandwich structure and SEM image of the PZT film upon the Al foil. f) Capacitance comparison between the PZT layer‐based tribolayer and the evenly dispersed PZT particle‐based tribolayer. g) SEM image of interweaved glass fiber bundles upon the PZT film. Reproduced with permission.^[^ ^8b^ ^]^ Copyright 2021, Wiley‐VCH. h) Electrical leakage state of the multilayered PVDF‐TrFE‐BT‐based TENG and the TENG with randomly dispersed BT nanoparticles. i) SEM image of a multilayered PVDF‐TrFE‐BT tribolayer. Reproduced with permission.^[^ ^24^ ^]^ Copyright 2020, American Chemical Society. j) Triboelectric series of common organic and inorganic dielectric tribo‐materials. k–r) Interface contact optimization effect of various MNIDMs on TENG outputs. SEM images of (k) 0D SiO_2_ nanoparticles, (l) 1D ZnO nanowires, (m) 2D ZnO nanowalls, (n) 3D TiO_2_ nanoflowers, (o) 3D ZnO nanoflowers, (p) the surface of ZnO nanoflower‐PDMS composite film, (q) electrospun PAN and (r) PAN‐B(OH)_3_ composite nanofibers. Reproduced with permission.^[^ ^63^ ^]^ Copyright 2013, American Chemical Society. Reproduced with permission.^[^ ^57^ ^]^ Copyright 2019, Elsevier. Reproduced with permission.^[^ ^64^ ^]^ Copyright 2017, Wiley‐VCH. Reproduced with permission.^[^ ^65^ ^]^ Copyright 2014, Wiley‐VCH.

From Equation (8), it can be concluded that the permittivity of tribo‐materials is positively correlated with the total transferred charge during a working cycle.^[^ ^66^ ^]^ Lots of IDMs are known for their ultrahigh permittivities, for example, TiO_2_ (*ɛ*_r_ = 80), BT (*ɛ*_r_ = 150), SrTiO_3_ (ST, *ɛ*_r_ = 300), PZT (*ɛ*_r_ = 1700), and CaCu_3_Ti_4_O_12_ (*ɛ*_r_ = 7500).^[^ ^9^, ^59^, ^67^ ^]^ Embedding various IDM micro‐/nanoparticles with high permittivities in the matrices of flexible tribo‐materials and constructing ultrathin IDM layers of this type are typical strategies for achieving high triboelectric outputs and ensuring device flexibility. Whichever strategy is chosen to prepare high‐permittivity IDM‐based tribo‐materials, simultaneously ensuring sufficient contact area of the tribo‐materials, reducing electric leakage, and improving surface trapping charge is key for optimizing device electrical outputs. In general, there are three approaches to meet the above‐mentioned targets: i) embedding nanoarchitectured high‐permittivity IDM (HPIDM) in nanoarchitectured polymer matrices, ii) fabricating HPIDM‐based sandwich structures, and iii) constructing HPIDM‐based nanocomposite multilayer structures. For example, Hu's and Tantraviwat's groups evenly dispersed high‐permittivity 10 wt% ST and 7 wt% BT particles, respectively, in microporous poly(dimethylsiloxane) (PDMS) matrices using the co‐blending method (**Figure** **3**a).^[^ ^59^, ^68^ ^]^ The porous microarchitectures enhance both compressibility and effective contact area, achieving high voltage, transferred charge density, and power density of 338 V, 19 nC cm^−2^, and 0.647 µW cm^−2^, respectively. In contrast, the low‐permittivity porous PDMS‐based TENG yielded outputs of only 257 V, 6.6 µA cm^−2^, and 13.2 nC cm^−2^.

The dispersity modulation of the HPIDM particles is also an important factor for the triboelectric output. Zhang et al. found that a TENG with uniformly distributed BT nanoparticles possesses higher permittivity, lower dielectric loss, and therefore higher electrical outputs (1020 V, 1.32 µA cm^−2^, and 5.8 nC cm^−2^) than an agglomerated IDM nanoparticle‐based TENG with the same BT content (780 V, 0.682 µA cm^−2^, and 3.7 nC cm^−2^, Figure 3b).^[^ ^60^ ^]^ Wen et al. synthesized a composite consisting of 1.5 wt% Ti_0.87_O_2_ sheets with a monolayer nanoarchitecture and poly(vinylidene fluoride) (PVDF) to elevate the tribocharge surface density at a certain HPIDM filler content. The high quantum size effect and large specific surface area of high‐permittivity Ti_0.87_O_2_ nanosheets led to good charge trapping capability, and therefore, a 50‐fold enhancement in output power density (92.5 µW cm^−2^) compared with that of a pure PVDF‐based TENG (Figure 3c).^[^ ^61^ ^]^ Shao et al. used a vacuum filtration method to introduce 13.5 vol% BT particles into a bacterial cellulose nanofiber film, which combined the enhancement of permittivity and optimization of the surface structure (Figure 3d). As a result, the transferred charge density increased from 3.44 nC cm^−2^ (for the sample without BT particles) to 7.52 nC cm^−2^.^[^ ^62^ ^]^ Zheng et al. sandwiched a high‐permittivity PZT layer between a bottom aluminum electrode and an upper glass‐fiber tribolayer (Figure 3e). The micron‐scale sandwich structure possessed a larger capacitance than the conventional composite layer comprising evenly dispersed IDM particles (Figure 3f). The HPIDM interlayer had no influence on the surface structure, which enabled a flexible surface micro‐/nanoarchitecture design to achieve a large effective contact area (Figure 3g). Based on these merits, the device had very high outputs of 1640 V, 10.29 nC cm^−2^, 5.905 µA cm^−2^, and 1080 µW cm^−2^.^[^ ^8b^ ^]^ Park et al. prepared a multilayer nanocomposite tribolayer with alternating poly(vinylidenefluoride‐co‐trifluoroethylene) (PVDF‐TrFE) and BT nanoparticle (6.5 wt%) layers, reducing the electric leakage among the BT nanoparticles (Figure 3h). Due to the reduced electric leakage, the multilayered structure provides superior interfacial polarization and boosted outputs of 44 V, 1.77 µA cm^−2^, and 29.4 µW cm^−2^.^[^ ^24^ ^]^ In contrast, remarkable electric leakage appeared in the composite tribolayer with randomly dispersed BT nanoparticles (Figure 3i), leading to low outputs of 35.4 V and 1.2 µA cm^−2^.^[^ ^24^ ^]^ Notably, it is essential to explore the optimum high‐permittivity IDM content. Insufficient IDM content cannot significantly improve the permittivity, while excessive IDM content will, in all probability, reduce the effective contact area or increase electric leakage.

#### 3.1.2 Boosting Triboelectric Polarity

From Equations (5) and (8), when the thickness of the dielectric tribolayer is much less than the distance between the two triboelectrically charged layers, *h*_0_ in Equation (8) can be ignored. In this case, the permittivity does not influence the transferred charge or tribocharge surface density, and the triboelectric polarity becomes one of the major factors dominating the output performance of TENGs. Compared to organic tribo‐materials, some IDMs such as mica, float glass, borosilicate glass, BeO and PZT‐5, possess excellent positive triboelectric polarity. After friction with other materials, these IDMs have edges that gain positive charges.^[^ ^8c^ ^]^ The triboelectric series is used to describe the relative strength of the triboelectric polarity of a material, i.e., the tendency of materials to generate triboelectric charges (Figure 3j).^[^ ^69^ ^]^ In general, when two materials rub against each other, the greater the distance between the two materials in the triboelectric series, the higher is the tribocharge surface density. Therefore, TENGs made of these IDMs with high positive triboelectric polarity and other tribo‐materials with negative triboelectric polarity could show high electrical outputs.^[^ ^12g^ ^]^ Mica is the most typical IDM with an incomparable ability to carry positive tribocharges (+61.8 µC m^−2^ with mercury as reference).^[^ ^8c^ ^]^ Notably, choosing suitable IDMs via the triboelectric series is an approximate strategy for obtaining a high TENG output. This is because the triboelectric series of IDMs was determined using mercury as a reference, the charge transfer of which is closely related to the work function and conforms to the quantum mechanical transition model.^[^ ^8c^ ^]^ However, when a pair of tribolayers does not contain metallic materials, there still exists other charge transfer mechanisms and resultant anomalous output performances that go against the supposition according to the triboelectric series. Furthermore, a common electrical performance enhancement strategy selects IDMs with high triboelectric polarities in conjunction with micro‐/nanoarchitectures for a large effective contact area.

#### 3.1.3 Interface Contact Optimization

The effective contact area for charge transfer is also an important factor that determines the output performance of TENGs owing to its close relationship with the tribocharge surface density. Many IDMs can be controllably processed and self‐assembled into materials with various micro‐/nanoarchitectures owing to their specific crystal structures and growth characteristics.^[^ ^4c^ ^]^ Using MNIDMs as tribo‐materials can directly or indirectly optimize the interface contact and improve the effective contact area of TENGs. The direct interface contact optimization strategies involve constructing micro‐/nanoarchitectured friction surfaces consisting entirely of 0D, 1D, 2D, and 3D IDMs (Figure 3k–n). Nanoparticles, nanowires, nanorods, nanowalls, nanobundles, nanoflowers, and their mixtures based on ZnO, SiO_2_, TiO_2_, zeolitic imidazole frameworks (ZIFs), Mo_6_S_3_I_6_, and perovskite are common 0D, 1D, 2D, and 3D IDMs that are used for the direct optimization of the effective contact area in the pressed state.^[^ ^8^, ^14^, ^26^, ^56^, ^57^, ^62^, ^63^, ^70^ ^]^ This is because the arrays of these nanoarchitectured materials can directly provide friction surfaces with high roughness. For example, Hong's group converted 2D ZnO nanowall configurations into yarns (Figure 3m), increasing the surface roughness and resultant the power density of the TENG from 13.7 µW cm^−2^ (corresponding to the bare yarn) to 68.5 µW cm^−2^.^[^ ^64^ ^]^ By wrapping the 2D nanowalls with 1D ZnO nanoparticles, the surface roughness was further increased, and therefore the power density increased to 629 µW cm^−2^.

In the case of a particularly low impact pressure, a friction surface with low roughness is easily in contact with other tribo‐materials and instead helps increase the effective contact area. Mica with sub‐nanometric roughness is a good example. Under a pressure as low as 1.7 kPa, the mica TENG with polytetrafluoroethylene (PTFE) as the opposite tribolayer delivers a boosted voltage of 147 V.^[^ ^70b^ ^]^ The indirect interface contact optimization strategy implies that IDMs are not directly exposed to the surface but are embedded in the organic matrices to form micro‐/nanoarchitectured friction surfaces, which can be categorized into the template type and the non‐template type. In template types, MNIDMs serve as skeletons while organic components serve as morphologically similar epidermises to contact other tribo‐materials. For example, Kim et al. fabricated a ZnO‐PDMS composite with ZnO nanoflowers as the skeleton and PDMS as the epidermis (Figure 3o,p). The rough composite surface endows the TENG with high electrical outputs of 400 V and 30 µA.^[^ ^26^ ^]^ In contrast, in non‐template types, MNIDMs do not act as templates but still change the surface structures of the organic tribo‐material. For example, the introduction of B(OH)_3_ nanoparticles into a polyacrylonitrile electrospinning solution increased the effective contact area of the TENG by decreasing the nanofiber diameter of the electrospun tribolayer (Figure 3q,r).^[^ ^70c^ ^]^

#### 3.1.4 Electron Trapping Effect

**Figure 4:**
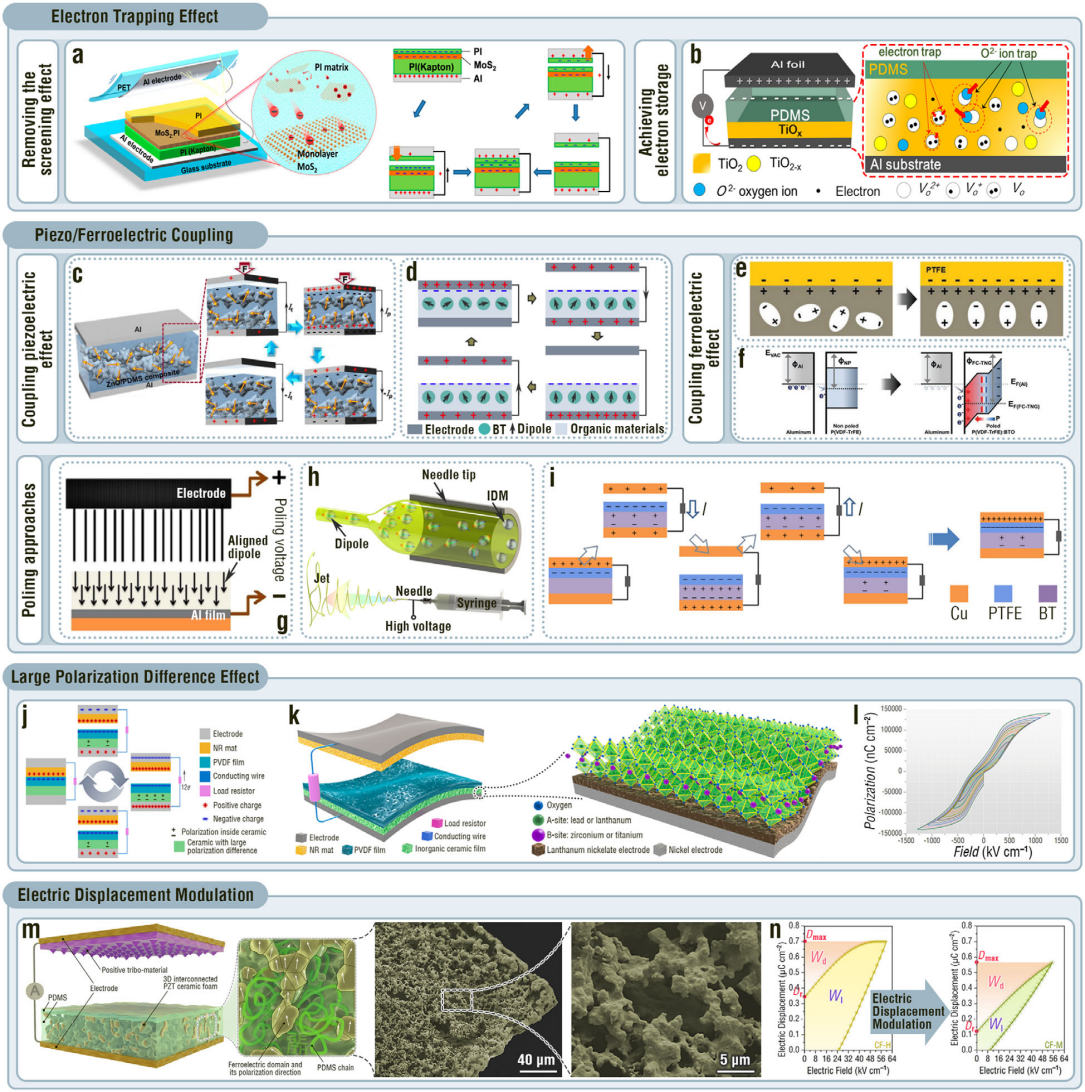
MNIDM‐based TENGs with electron trapping effect, piezo/ferroelectric coupling effect, large polarization difference effect, and electric displacement modulation effect. a,b) Electron trapping effect of MNIDM‐based TENGs. a) Schematic illustration and working mechanism of the MoS_2_‐based TENG with electron trapping effect. Reproduced with permission.^[^ ^71b^ ^]^ Copyright 2017, American Chemical Society. b) Schematic illustration of the TiO*x*‐based TENG with electron trapping effect. Reproduced with permission.^[^ ^72^ ^]^ Copyright 2018, Elsevier. c–i) Piezo/ferroelectric coupling effect of MNIDM‐based TENGs. c) Schematic illustration of the working mechanism for the TENG based on inorganic non‐ferroelectric piezoelectrics. Reproduced with permission.^[^ ^26^ ^]^ Copyright 2018, American Chemical Society. d) Schematic illustration of the working mechanism for the TENG based on inorganic ferroelectric piezoelectrics. e) Schematic illustration of the mechanism of enhancement in surface charge generation by aligned dipoles in the inorganic ferroelectric nanoparticles filled into conductive tribo‐materials. Reproduced with permission.^[^ ^73^ ^]^ Copyright 2019, Wiley‐VCH. f) Schematic illustration of the mechanism for the TENG with the dielectric tribo‐material‐inorganic ferroelectric composite and the metal serving as the tribolayers. Reproduced with permission.^[^ ^74^ ^]^ Copyright 2016, Wiley‐VCH. Schematic diagrams of (g) the post poling, (h) in situ poling, and (i) triboelectricity poling. Reproduced with permission.^[^ ^75^ ^]^ Copyright 2017, IOP Publishing. Reproduced with permission.^[^ ^35^ ^]^ Copyright 2019, Royal Society of Chemistry. Reproduced with permission.^[^ ^52b^ ^]^ Copyright 2017, Springer Nature. j–l) Large polarization difference effect of MNIDM‐based TENGs. j) Schematic illustration of the working mechanism of the TENG using an inorganic dielectric ceramic with large polarization difference as the interlayer. k) Schematic diagram of the structure for the Pb_0.94_La_0.04_Zr_0.98_Ti_0.02_O_3_‐based TENG with large polarization difference effect. l) Polarization‐electric field loops of the Pb_0.94_La_0.04_Zr_0.98_Ti_0.02_O_3_ antiferroelectric ceramic film. Reproduced with permission.^[^ ^9b^ ^]^ Copyright 2021, Elsevier. m,n) Electric displacement modulation effect of MNIDM‐based TENGs. m) Schematic diagram and SEM images of the TENG based on submicron interconnected PZT skeletons. n) Electric displacement‐internal electric field loops of the PDMS‐filled submicron interconnected PZT skeleton before and after electric displacement modulation. *W*_d_ is the recoverable energy‐storage density. *W*_l_ is the energy loss density. Reproduced with permission.^[^ ^76^ ^]^ Copyright 2024, Cell Press.

The output performance of a TENG is highly related to the tribocharge density.^[^ ^52b^ ^]^ In practical applications, the tribocharge density decays with time owing to the adsorption of charged ions or particles from the air and their combination with induced charges on the electrode.^[^ ^52^, ^71^ ^]^ Moreover, the screening of induced charges by interfacial electrons between the tribonegative layer and electrode degrades the tribocharge density.^[^ ^72^ ^]^ Some nanoarchitectured IDMs featuring excellent electron trapping effect are suitable fillers to help the TENG maintain tribocharges at a high level. 2D monolayer MoS_2_ is a good example and can serve as a triboelectric electron‐acceptor layer owing to its large specific surface area, quantum confinement effect, and large intrinsic bandgap energy of 1.8 eV.^[^ ^71b^ ^]^ In the working process of the MoS_2_‐based TENG, a large number of electrons are continuously stored in the monolayer MoS_2_, leading to a high potential difference and, therefore, enhanced outputs (**Figure** **4**a). Compared with the device without the MoS₂ layer, the performances increased from 30 V, 1.6 µA cm^−2^, and 13.3 nC cm^−2^ to 400 V, 3.93 µA cm^−2^, and 53.3 nC cm^−2^. In addition, introducing a 100‐nm‐thick TiO_x_ electron‐blocking layer (EBL) between the tribonegative layer and an electrode can enhance the tribocharge density by removing the screening effect from the interfacial electrons in the bottom electrode to the induced positive charges at the interface beneath the tribonegative layer.^[^ ^72^ ^]^ This is because the oxygen vacancies and charge transport properties of the TiO_x_ EBL can block electrons (Figure 4b). The optimum TiO_x_ thickness was determined according to the trade‐off between the electron‐blocking properties and the total capacitance of the tribonegative layer. As a consequence, the voltage and current density drastically increased from 50 V and 0.222 µA cm^−2^ to 272 V and 1.01 µA cm^−2^, respectively.

#### 3.1.5 Piezo/Ferroelectric Coupling

IDMs typically exhibit excellent piezoelectricity and ferroelectricity. These two types of effects can couple with the triboelectric effect, enhancing the surface charge density and, therefore, the electrical output.^[^ ^26^, ^52^, ^73^, ^74^, ^75^ ^]^ Due to the similar output characteristics, working frequency, and matched resistance of triboelectric and piezoelectric devices,^[^ ^76^ ^]^ directly combining the triboelectric and piezoelectric effects enhances the electrical output.^[^ ^77^ ^]^ Embedding inorganic piezoelectric nanoparticles into matrices of organic tribo‐materials is the most common approach for coupling these two effects (Figure 4c,d).^[^ ^75^ ^]^ This enabled the external pressure to simultaneously alter the triboelectric field and piezoelectric polarization in the device, resulting in a coupled output. Inorganic piezoelectric materials exhibit remarkable piezoelectric responses.^[^ ^78^ ^]^ Organic materials (e.g., PDMS, Figure 3j) are responsible for strongly trapping surface tribocharges.^[^ ^36^ ^]^ Because contact electrification is an interface effect reliant on surface tribo‐materials, whereas piezoelectricity is a bulk effect,^[^ ^1g^ ^]^ replacing part of the internal tribo‐materials with inorganic piezoelectric materials introduces piezoelectric effects without weakening them.^[^ ^4b^ ^]^ In addition, nano‐sized embedded inorganic piezoelectric particles are easily dispersed evenly in organic matrices, ensuring both optimal binary‐coupled electrical output and good device flexibility.

To couple the ferro‐ and triboelectric effects, inorganic ferroelectric micro‐/nanoparticles and films are typically used as fillers or interlayers, with tribo‐materials acting as matrices or surface layers.^[^ ^4^, ^52^, ^73^, ^74^ ^]^ The hysteretic polarization of ferroelectrics can induce more net surface tribocharges.^[^ ^52b^ ^]^ Coupling effects can occur regardless of whether inorganic ferroelectric materials are filled into conductive or dielectric tribo‐materials. When inorganic ferroelectric nanoparticles are filled into conductive tribo‐materials, the aligned dipoles in the ferroelectrics induce a net electric field that can lead to a larger charge transfer during contact electrification (Figure 4e).^[^ ^73^ ^]^ For semiconductor and metal triboelectric materials, a powerful ferroelectric polarization induces a Fermi level shift and increased charge transfer (Figure 4f).^[^ ^74^ ^]^ In general, coupling the ferroelectric effect results in greater performance enhancement than solely coupling the piezoelectric effect. For example, Seung et al. developed a PZT−PVDF‐TrFE nanocomposite material to couple the triboelectric and ferroelectric effects, achieving an 11‐fold increase in the output voltage (330 V) compared to the pure triboelectric output (28.4 V).^[^ ^74^ ^]^ In contrast, the triboelectric–piezoelectric coupling output does not exceed twice the sum of the individual effects.^[^ ^26^ ^]^

It is worth noting that the coupling effect is activated only when the inorganic ferroelectrics are electrically poled and the dipole direction coincides with the triboelectric polarization direction. There are three common poling approaches: i) post‐poling, ii) in situ poling, and iii) triboelectricity poling. Post‐poling means that the pristine sample underwent a poling treatment using an external high‐voltage source (Figure 4g).^[^ ^75^ ^]^ In situ poling occurs during the processes of sample preparation, such as electrospinning and electrostatic atomization. After the sample is fabricated, it is endowed with macroscopic polarity (Figure 4h).^[^ ^4b^ ^]^ Triboelectric poling depends on the electric field between the tribolayer surface and the electrode in the separation state of the TENG. After a certain number of working cycles, the inorganic ferroelectrics in the tribolayer are fully polarized (Figure 4i).^[^ ^52b^ ^]^

#### 3.1.6 Large Polarization Difference Effect

The amount of transferred charge through the external circuit is one of the most important parameters of a TENG because it directly reflects the device output performance and the available electric energy stored in external capacitors. Achieving a high transferred charge density requires not only a high surface charge density on the dielectric tribolayer but also a low charge density on the electrode attached to the dielectric tribolayer in the pressed state to enable more charges to flow through the external circuit.^[^ ^9^, ^79^ ^]^ Inserting an inorganic dielectric layer with a large polarization difference (i.e., the value of the maximum polarization minus the remnant polarization) between the electrode and tribolayer is ideal.^[^ ^9^, ^80^ ^]^ In the separated state, there is a triboelectric potential difference between the dielectric tribolayer surface and its electrode, which interacts with the inserted inorganic dielectric layer (Figure 4j). Consequently, the inserted inorganic dielectric layer is polarized, and the tribocharge surface density is enhanced. The dielectric polarization, surface charge density, and charge density in the electrode simultaneously reached their maximum values in the released state. When the TENG returned to the pressed state, there was no difference in the triboelectric potential between the dielectric tribolayer surface and its electrode. Both dielectric polarization and charge density in the electrode were reduced to their minimum values (preferably zero). When the TENG operates circularly between the released and pressed states, a large amount of charge in the electrode is output to the external circuit owing to the large polarization difference effect. Because the triboelectric potential difference is relatively low (approximately several hundred to several thousand volts), the appropriate thickness of the inserted inorganic dielectric layer should range from a few hundred nanometers to a few micrometers.^[^ ^9b^ ^]^ Zhang et al. synthesized a 1.35‐µm‐thick Pb_0.94_La_0.04_Zr_0.98_Ti_0.02_O_3_‐based inorganic dielectric interlayer with a record‐high polarization difference of 1065 mC m^−2^ at 1300 kV cm^−1^ (Figure 4k,l), endowing the TENG with a 2‐fold enhancement in transferred charge density (13.5 nC cm^−2^).^[^ ^9b^ ^]^

#### 3.1.7 Electric Displacement Modulation

As discussed in Section 2, a TENG can be abstracted as a dielectric capacitor. Therefore, a strategy for enhancing the performance of the dielectric capacitor can be used to improve the output of a TENG. Modulating the relationship between the electric displacement and the electric field of MNIDMs is an important approach for enhancing the recoverable energy storage density of dielectric capacitors.^[^ ^81^ ^]^ Recently, Zhang et al. developed a similar electric displacement‐internal electric field modulation strategy to enhance the performance of TENGs.^[^ ^76^ ^]^ By elaborating a tribo‐material consisting of PDMS‐filled submicron‐interconnected PZT skeletons (Figure 4m), the electric displacement–internal electric field loop was significantly reduced, and the dielectric loss of the tribo‐material was minimized, endowing the tribo‐material with a high recoverable energy storage density (Figure 4n). In the tribo‐material, the continuous distribution of the interconnected PZT microskeleton guarantees a strong polarization response, and the incomplete filling of the PZT decreased the polarization response difference in the material charging and discharging processes. In addition, the PDMS filling prevented the PZT skeleton from breaking during mechanical deformation, ensuring the robustness of the device. The increased recoverable energy storage density of the materials further enhanced the discharge energy density of the TENG. As a result, the electric‐displacement‐modulated TENG based on submicron interconnected PZT skeletons exhibited a high discharge energy density of 2.76 µJ cm^−2^, a 330% improvement compared with the device without the electric displacement modulation effect.

In brief, the micro‐/nanoarchitectures of the various IDMs significantly enhanced the electrical performance of TENGs. The practical application of these strategies are attracting increasing attention. All‐inorganic dielectric tribolayers offer excellent long‐term storage stability, but entail high synthesis costs per unit area and challenges in large‐scale production. Their high rigidity also increases the risk of fracture with prolonged use. In contrast, organic–inorganic composite structures provide better flexibility, lower fracture risks during long‐term use, and easier large‐scale fabrication. However, excessive IDM content or layered IDM structures may lead to interface rupture or delamination owing to the modulus mismatch between the organic and inorganic materials. Therefore, the MNIDM design should balance electrical performance and practicality.

### 3.2 PENGs Based on MNIDMs

Inorganic piezoelectrics are known for their outstanding electrical outputs and excellent performance stability under extreme conditions, such as high temperatures,^[^ ^82^ ^]^ compared with organic piezoelectrics.^[^ ^12^, ^83^ ^]^ However, typical inorganic piezoelectrics are inflexible oxides that undergo brittle deformation, which limits their applications in biomechanical energy harvesting and wearable sensing.^[^ ^84^ ^]^ The construction of special micro‐/nanostructures can bestow inorganic piezoelectric materials with flexibility. The available methods are divided into two types: synthesis of 1D^[^ ^85^ ^]^ or 2D inorganic piezoelectrics^[^ ^9^, ^52^, ^86^ ^]^ and preparation of organic–inorganic composites. Common composite styles include 0–3,^[^ ^17^, ^87^ ^]^ 1–3,^[^ ^19^, ^88^ ^]^ 2–2,^[^ ^18^, ^89^ ^]^ and 3–3^[^ ^15^ ^]^ composites. For example, Lee's group fabricated a 2‐µm‐thick 2D PZT film to endow a corresponding PENG with flexibility.^[^ ^52a^ ^]^ The device with a working area of 1.5 × 1.5 cm^2^ can be bent more than 3000 times at a bending radius of 1.61 cm. More importantly, multiple effects from inorganic piezoelectrics coupling micro‐/nanoarchitectures contribute to piezoelectric performance enchantment, which typically include i) permittivity optimization, ii) constructing local heterogeneity, iii) inserting symmetry‐breaking defects, vi) boosting load‐transfer efficiency, v) piezoelectric and semiconducting property coupling, vi) weakening screening effect, vii) poling effect enhancement, viii) electret/flexoelectric coupling, and ix) constructing multiple boundary interfaces.^[^ ^15^, ^17^, ^19^, ^45^, ^53^, ^87^, ^90^, ^91^, ^92^, ^93^, ^94^, ^95^, ^96^, ^97^, ^98^, ^99^, ^100^, ^101^, ^102^, ^103^, ^104^, ^105^, ^106^, ^107^, ^108^, ^109^, ^110^ ^]^

#### 3.2.1 Permittivity Optimization

Based on Equation (17), the figure of merit (*FOM*) of a PENG (i.e., the quantity used to describe the ability of materials to generate energy for practical applications) can be expressed as follows:

(21) $$FOM=\frac{d_{\mathrm{ij}}^{2}}{\varepsilon_{0}\varepsilon_{r}}$$

**Figure 5:**
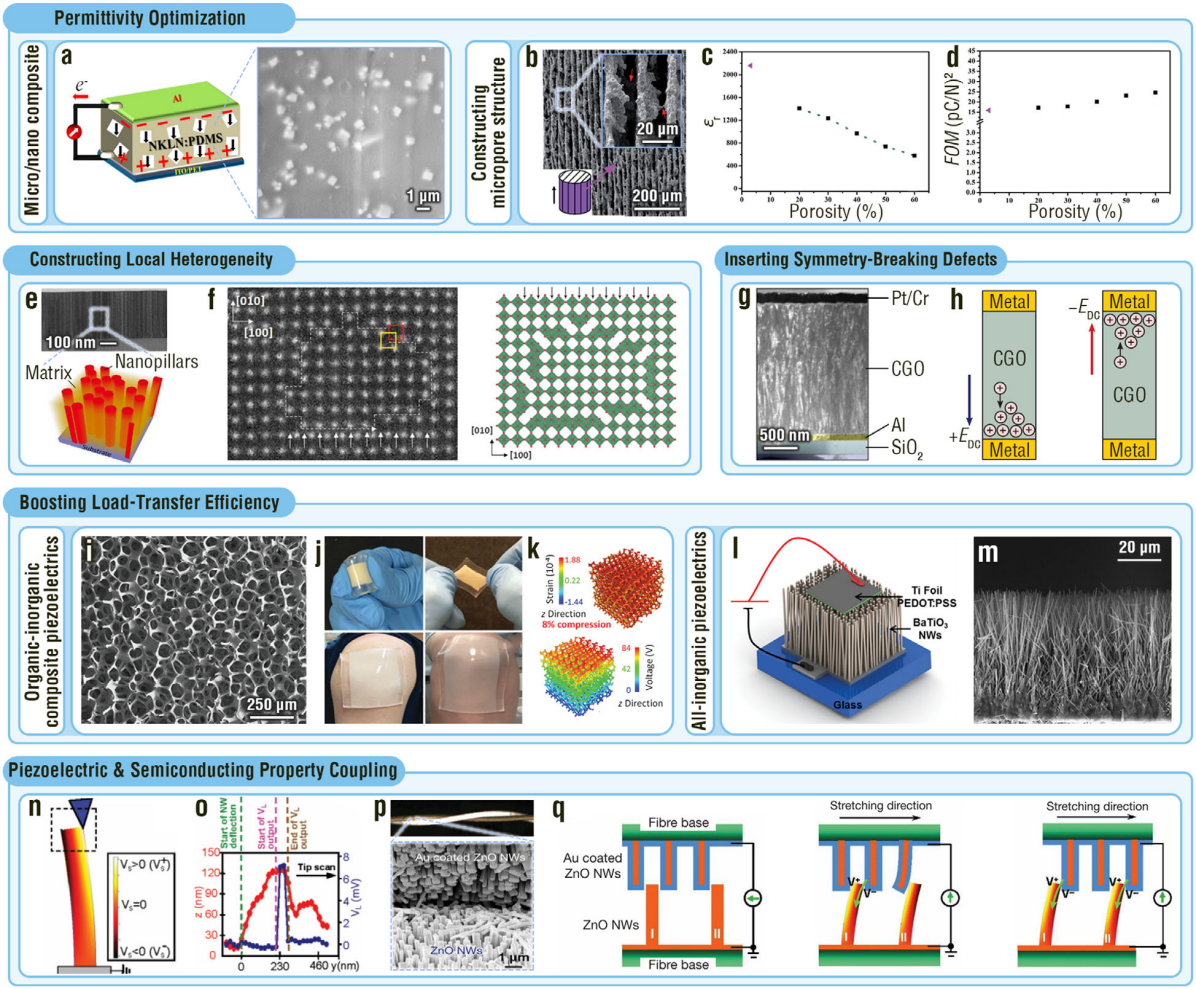
MNIDM‐based PENGs with optimized permittivity, local structural heterogeneity, symmetry‐breaking defects, boosted load‐transfer efficiency, and coupled piezoelectric and semiconducting properties. a–d) Permittivity optimization effect of MNIDM‐based PENGs. a) Schematic diagram and SEM image of a nanocomposite PENG consisting of NKLN microcubes and PDMS polymer. Reproduced with permission.^[^ ^17^ ^]^ Copyright 2018, Royal Society of Chemistry. b) Schematic diagram and SEM image of a porous parallel‐connected PZT piezoelectric ceramic. c) Dependence between the permittivity and the porosity of the porous parallel‐connected PZT piezoelectric ceramic. d) Dependence between the *FOM* and the porosity of the porous parallel‐connected PZT piezoelectric ceramic. Reproduced with permission.^[^ ^91^ ^]^ Copyright 2017, Royal Society of Chemistry. e,f) Local heterogeneity triggering effect of MNIDM‐based PENGs. e) Scanning transmission electron microscopy image and structure schematic of NPR‐NNO. f) Plan‐view atomically resolved image and corresponding structural models of the NPR‐NNO film. Reproduced with permission.^[^ ^93^ ^]^ Copyright 2020, American Association for the Advancement of Science. g,h) Inserting symmetry‐breaking defects of MNIDM‐based PENGs. g) SEM image of the CGO thin film. h) Schematic diagram showing the electric field‐driven oxygen vacancy migration. Reproduced with permission.^[^ ^45^ ^]^ Copyright 2022, American Association for the Advancement of Science. i–k) Load‐transfer efficiency boosting effect of MNIDM‐based PENGs. i) SEM image of the 3D interconnected PZT ceramic microfoam. j) Optical images showing the mechanical flexibility of the 3D composites. k) Finite element simulation showing the strain field and electric potential of the 3D interconnected PZT ceramic microfoam when the composite is subjected to 8% compressive strain. Reproduced with permission.^[^ ^15a^ ^]^ Copyright 2018, Royal Society of Chemistry. l) Schematic diagram of the PENG based on high aspect ratio BT nanowires. m) SEM image of vertically aligned BT nanowire arrays. Reproduced with permission.^[^ ^99^ ^]^ Copyright 2014, Wiley‐VCH. n–q) Piezoelectric–semiconducting properties coupling effect of MNIDM‐based PENGs. n) Potential distribution in the ZnO nanowire as a result of the piezoelectric effect. o) Line profiles from the topography (red) and output voltage (blue) images across a ZnO nanowire. p) Optical micrograph of a pair of entangled fibers and SEM image at the “teeth‐to‐teeth” interface of two fibers covered by nanowires. q) Working mechanism of the PENG based on a pair of entangled fibers wrapped by ZnO nanowire. Reproduced with permission.^[^ ^102^ ^]^ Copyright 2008, American Association for the Advancement of Science.

It was concluded that decreasing the permittivity is beneficial for improving the piezoelectric performance. In addition to using inorganic low‐permittivity piezoelectrics such as alkaline niobate (NKLN, with a permittivity ranging from 450 to 660),^[^ ^90^ ^]^ micro‐/nanoarchitecture is an effective and sophisticated strategy for optimizing the permittivity of IDM‐based piezoelectrics. Because the permittivities of both air and organic materials are significantly lower than those of inorganic piezoelectrics, micro‐/nanocomposites and the construction of micropore structures are the two main methods. In a specific operation, it is necessary to explore the optimal inorganic filler content and porosity to obtain a high *FOM* because the piezoelectric coefficient is also influenced by micro‐/nanoarchitecture engineering. For example, Gupta et al. prepared a piezoelectric composite consisting of Na_0.47_K_0.47_Li_0.06_NbO_3_ microcubes and a PDMS polymer in a volume ratio of 4:6 (**Figure** **5**a).^[^ ^17^ ^]^ The low permittivity of this composite resulted in a high electromechanical coupling factor and outstanding energy conversion efficiency of up to 11.12%. Jeong et al. also embedded NKLN particles in PDMS, achieving the piezoelectric outputs of 12 V and 0.13 µA cm^−2^ at the optimum NKLN content. As the NKLN content continued to increase, the outputs remained at only 3.4 V and 0.022 µA cm^−2^ due to the excessively high permittivity.^[^ ^90a^ ^]^ Zhang et al. fabricated a porous parallel‐connected PZT piezoelectric ceramic with a porosity of 60% via freeze casting (Figure 5b).^[^ ^91^ ^]^ The introduction of parallel‐aligned micropore structures reduced the permittivity (Figure 5c), which had beneficial consequences for the *FOM* and, therefore, piezoelectric performance (Figure 5d).

#### 3.2.2 Constructing Local Heterogeneity

According to Equation (21) and the discussion in Section 2.2, a high piezoelectric coefficient indicates an outstanding electrical output of the PENG. Local structural heterogeneity at the nanoscale plays an important role in realizing ultrahigh piezoelectric coefficients.^[^ ^92^ ^]^ Meanwhile, inorganic piezoelectrics typically exhibit inherently high piezoelectric coefficients. Therefore, achieving local heterogeneity in inorganic piezoelectric materials by means of special micro‐/nanoarchitectures will further give rise to remarkably enhanced piezoelectric coefficients. Yao et al. fabricated Na‐deficient NaNbO_3_ (NNO) with nanopillar regions (NPR‐NNO) by sputter deposition (Figure 5e).^[^ ^93^ ^]^ The size of the vertical nanopillars ranged from a few to tens of nanometers. The nanopillars were embedded in the matrix of a regular perovskite structure (Figure 5e). Inside the NPRs, the Nb atoms occupied the original Na positions along both the in‐plane directions, whereas the Na atoms occupied the original Nb positions along both the in‐plane directions (Figure 5f). The structural distortions around the NPRs led to a reduction in crystal symmetry from tetragonal to monoclinic structure, promoting polarization rotation and domain wall motion under electric fields to considerably boost piezoelectric coefficient *d*_33_ from 22 to 1098 pm V^−1^.^[^ ^93^ ^]^

#### 3.2.3 Inserting Symmetry‐Breaking Defects

The insertion of symmetry‐breaking defects has also been demonstrated to be an effective strategy for achieving high piezoelectric coefficients, even in centrosymmetric crystals.^[^ ^94^ ^]^ When point defects are inserted in a material, an external electric field can be used to manipulate them, breaking the symmetry of the crystal and enhancing its piezoelectricity.^[^ ^95^ ^]^ Park et al. used a high direct current electric field of 1 MV cm^−1^ on a centrosymmetric 1.8‐µm‐thick CGO thin film (Figure 5g) to migrate oxygen vacancies (i.e., point defects), which leads to symmetry breaking and thus incorporating piezoelectricity in the film (Figure 5h).^[^ ^45^ ^]^ Astonishingly, the induced piezoelectric coefficient is as high as 200 000 pm V^−1^. This strategy can be extended to other IDMs such as yttria‐stabilized zirconia to achieve competitive piezoelectric responses. Notably, this type of piezoelectric material has the unparalleled advantage of withstanding ultrahigh pressures (>250 MPa) without de‐poling.^[^ ^46^ ^]^ In light of these advantages, CGO‐based PENGs are expected to enable efficient biomechanical energy harvesting and highly sensitive pressure sensing over a wide dynamic range.

#### 3.2.4 Boosting Load‐Transfer Efficiency

As the strain is closely associated with the applied stress *T*, according to Equations ((16), (17), (18)), increasing the strain acting on the piezoelectrics can also enhance the voltage, current, and energy. Boosting the load‐transfer efficiency by processing IDMs into various micro‐/nanostructured piezoelectrics, such as microspheres,^[^ ^96^ ^]^ microhemispheres,^[^ ^21^ ^]^ microclusters,^[^ ^23^ ^]^ microspindles,^[^ ^97^ ^]^ microflowers,^[^ ^98^ ^]^ microwires,^[^ ^15^, ^99^ ^]^ micropores,^[^ ^100^ ^]^ and microfoams,^[^ ^15b^ ^]^ is an effective approach for increasing the strain and therefore achieving high electrical outputs.^[^ ^101^ ^]^

In organic–inorganic composites, there is a large stiffness disparity between the inorganic filler and the polymer matrix of the piezoelectric composite. The 0–3 composite is the most common organic–inorganic piezoelectric composite. In this mode, the spatial discontinuity of the IDM phase results in poor load transfer from the surrounding polymer matrix to the active inorganic piezoelectric filler, leading to low piezoelectric outputs. In contrast, a 3D continuous inorganic piezoelectric filler across the top and bottom surfaces of the composite can provide highly effective load transfer, independent of the relative stiffness of the material's constituents. For example, Wang et al. prepared a flexible 3D interconnected PZT ceramic foam‐based piezoelectric composite (Figure 5i,j).^[^ ^15a^ ^]^ The design of a 3D interconnected microfoam structure increases load‐transfer efficiency by a factor of 17 500 in comparison with the 0–3 composite piezoelectric material made from PZT nanoparticles, increasing the outputs from 7 V and 0.4 nA to 85 V and 40 nA, respectively (Figure 5k).

For all‐inorganic piezoelectrics, the micro‐/nanostructural design has a significant impact on the output. Typically, high‐aspect‐ratio nanowires can exhibit relatively high deformations to produce an enhanced piezoelectric response at a lower stress level. Koka et al. fabricated a PENG (see Figure 5l) made from ultralong, vertically aligned BT nanowire arrays (the nanowire with a length of 40 µm and a diameter of 630 nm, see Figure 5m).^[^ ^99^ ^]^ Under an input root mean square base acceleration of only 0.25 g, the PENG delivered high outputs of 0.775 V, 1.86 nA, and 192 nW cm^−2^.

#### 3.2.5 Piezoelectric and Semiconducting Property Coupling

The first PENG based on ZnO nanowires was known for its high mechanical‐to‐electrical energy conversion efficiency of 17%–30% (Figure 5n).^[^ ^6^ ^]^ Its high electrical performance relies on piezoelectric and semiconducting property coupling,^[^ ^102^, ^103^ ^]^ which dominates the processes of charge creation, accumulation, and release, and delivers direct current signals (Figure 5o).^[^ ^104^ ^]^ More importantly, the coupling effect between the piezoelectric and semiconducting properties achieves the constructive accumulation of the piezoelectric output from each ZnO nanowire by means of a simple device structure design. This is vital for efficient conversion of biomechanical energy into electrical energy. Assuming that piezoelectric ZnO nanowire arrays do not have semiconductor properties, current signals in different directions may cancel each other out unless complex circuits are built to manage these myriad electrical signals. In contrast, the Schottky barrier stemming from the piezoelectric and semiconducting property coupling effect can prevent the flow of charge based on a reverse‐biased Schottky contact, but promotes charge movement in the forward‐biased Schottky contact state (Figure 5p,q).^[^ ^104^ ^]^ Therefore, all piezoelectric outputs of ZnO nanowire arrays sandwiched between two electrodes can be summated and reach a high level to meet the requirement in practical applications. Micro‐/nanoarchitectured fibers and fabrics based on ZnO nanowire arrays can even be easily produced to effectively scavenge biomechanical energy. Wang et al. fabricated ZnO nanowires that grew radially around micron‐scale textile fibers (Figure 5p).^[^ ^102^ ^]^ By entangling two fibers and brushing the nanowires rooted on them with respect to each other, body‐movement energy was continuously converted into electricity, regardless of the pulling and deflection directions of the fibers and ZnO nanowires, owing to the piezoelectric and semiconductor property coupling effect (Figure 5q).

#### 3.2.6 Weakening Screening Effect

**Figure 6:**
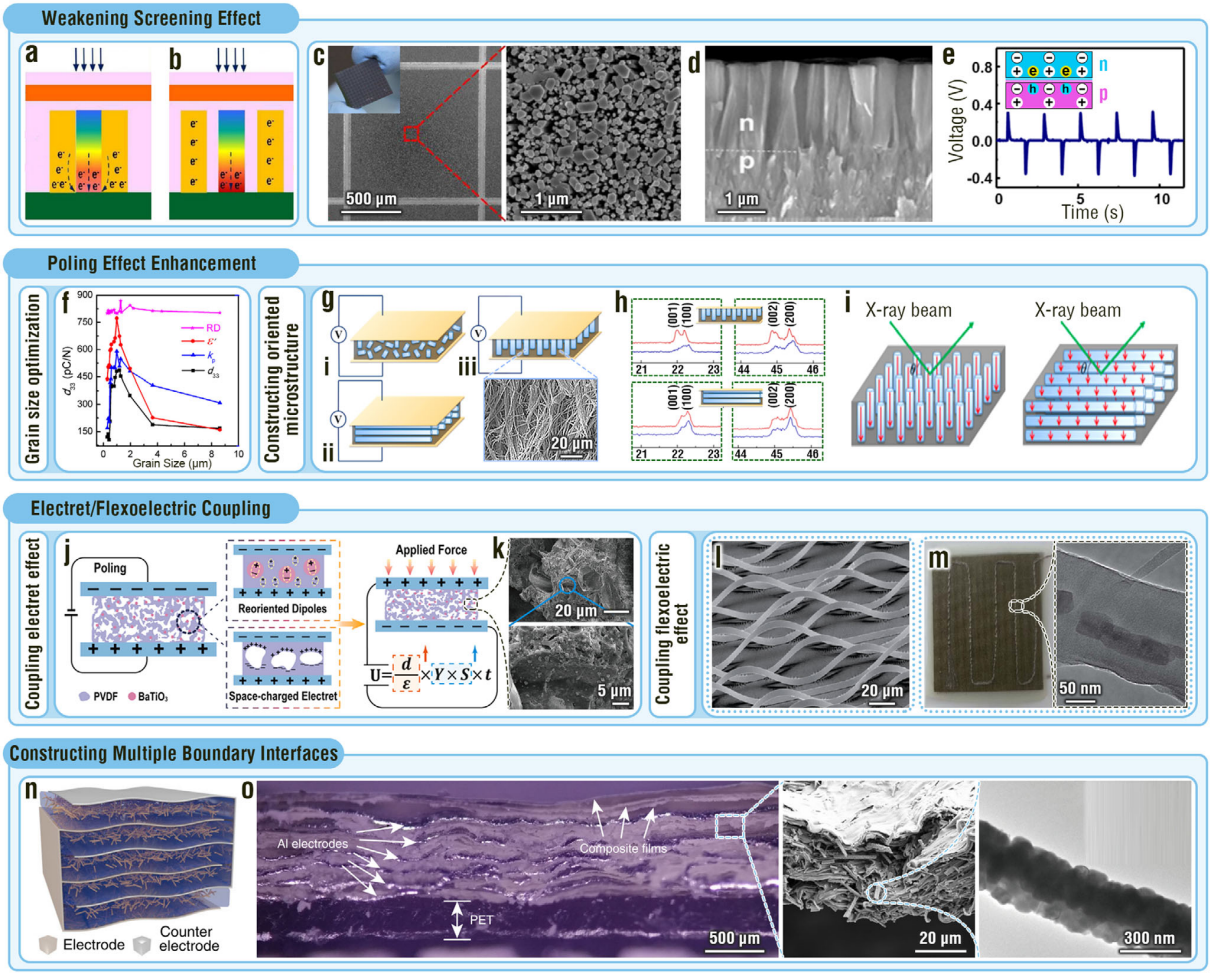
MNIDM‐based PENGs with diminished screening effect, enhanced poling effect, electret/flexoelectric coupling effect, and multi‐boundary interface effect. a–e) Diminished screening effect of MNIDM‐based PENGs. a) Schematic diagram showing the screening effect without segmentation. b) Schematic diagram showing the diminished screening effect thanks to the segmentation structure. Reproduced with permission.^[^ ^105^ ^]^ Copyright 2012, American Chemical Society. c) Photograph and SEM images showing the nanowire arrays segmented into isolated microzones. d) SEM image of the n−p homojunction of two layers of ZnO nanowire arrays. e) Removing screening effect mechanism and output voltage of the PENG based on n−p homojunction. Reproduced with permission.^[^ ^53a^ ^]^ Copyright 2014, American Chemical Society. f–i) Enhanced poling effect of MNIDM‐based PENGs. f) Piezoelectric coefficient *d*_33_ versus grain size of the BT ceramic. Reproduced with permission.^[^ ^106^ ^]^ Copyright 2014, Elsevier. g) Schematic diagram of BT nanofiber–PDMS composite‐based PENG with three kinds of nanofiber alignment modes ((i) random, (ii) horizontal, and (iii) vertical alignments) and SEM image of BT nanofibers aligned along the poling field direction. h) X‐ray diffraction patterns of the two PENGs with BT nanofibers respectively aligned along and perpendicular to the poling field direction before (blue lines) and after poling (red lines). i) Schematic diagram showing the poling process for the BT nanofiber–PDMS composites with two different nanofiber alignment modes. Reproduced with permission.^[^ ^19^ ^]^ Copyright 2016, American Chemical Society. j–m) electret/flexoelectric coupling effect of MNIDM‐based PENGs. j) Working mechanism of the PENG with a hierarchical porous PVDF foam embedded with BT nanoparticles. k) SEM image of the BT nanoparticle–PVDF composite. Reproduced with permission.^[^ ^107^ ^]^ Copyright 2022, Elsevier. l) SEM image of buckled PZT ribbons. Reproduced with permission.^[^ ^108^ ^]^ Copyright 2011, American Chemical Society. m) Photograph and TEM image of the PENG consisting of a PVDF‐NaNbO_3_ nanofiber nonwoven fabric. Reproduced with permission.^[^ ^109^ ^]^ Copyright 2013, Royal Society of Chemistry. n,o) Multiple boundary interfaces of MNIDM‐based PENGs. n) Schematic diagram of the PENG with the 3D intercalation electrode. o) Optical micrograph, SEM, and TEM images of the PENG consisting of the 3D intercalation electrode and the Sm‐PMN‐PT nanowire–PDMS composite. Reproduced with permission.^[^ ^87h^ ^]^ Copyright 2020, Springer Nature.

ZnO nanowire array‐based PENGs provide excellent opportunities for low‐frequency biomechanical energy scavenging and physiological information recognition.^[^ ^6^, ^102^, ^103^, ^104^ ^]^ However, the screening effect of free carriers within the ZnO nanowires impairs the piezoelectric output. Arranging ZnO nanowire arrays using micro‐/nanoarchitectures is a key approach for minimizing the screening effect. When ZnO nanowire arrays are subjected to unevenly distributed pressure, the nanowires located beneath the force application area yield a piezopotential, whereas free carriers in the adjacent unstressed nanowires tend to drift toward the stressed nanowires to screen the piezopotential (**Figure** **6**a). Segmenting nanowire arrays into isolated microzones can segregate free carriers from adjacent unstressed nanowires to attenuate the screening effect (Figure 6b,c). Consequently, the piezoelectric outputs increased from 37 V and 12 µA cm^−2^ (for nanowire arrays that are not segmented into microzones) to 58 V and 134 µA cm^−2^, respectively.^[^ ^105^ ^]^ Furthermore, even when the ZnO nanowire arrays were under even pressure, the screening effect within each nanowire remained. To solve this problem, Wang's group investigated the influence of the deposition structure of n‐ and p‐ZnO on the piezoelectric performance.^[^ ^53a^ ^]^ When 1.5‐µm‐thick n‐ZnO was deposited onto p‐ZnO with the same thickness (Figure 6d), the free electrons and holes in the n‐ and p‐type layers, respectively, were attracted toward the n−p homojunction, making the piezoelectric polarization charges at the electrodes unscreened (Figure 6e). Therefore, the PENG outputs surged from 0.0071 V and 0.415 nA cm^−2^ (for a single n‐type sample) to 0.284 V and 2.65 nA cm^−2^, respectively.

#### 3.2.7 Poling Effect Enhancement

As discussed in Section 2.2, electrical poling is essential for triggering piezoelectricity in inorganic ferroelectrics. Therefore, the poling effect directly determines the ultimate piezoelectric output. The micro‐/nanostructures of inorganic ferroelectrics can significantly influence the poling effect. The grain size is a determinant—only the optimum grain size resulted in the best poling effect and piezoelectric output (Figure 6f).^[^ ^106^ ^]^ Oversized grains (more than a few microns) of piezoelectric ceramics can reduce the breakdown strength, while a grain size that is too small (less than several hundred nanometers) can restrict the ferroelectric domain density, thereby degenerating the poling effect.^[^ ^110^ ^]^ Moreover, designing the microstructure of ferroelectrics and piezoelectrics on scales beyond their grain sizes can enhance the poling effect. For example, Yan et al. found that in BT nanofiber–PDMS composite piezoelectrics, the composite with BT nanofibers aligned along the poling field direction had the best poling effect (demonstrated by the highest (001) and (002) diffraction peak intensities) compared to other arrangements (disordered or perpendicular to the poling field direction) (Figure 6g,h).^[^ ^19^ ^]^ This is because a larger electric field was imposed on the BT nanofibers aligned along the poling direction (Figure 6i). As a result, the outputs of the sample with BT nanofibers aligned along the poling field direction reached 2.67 V, 0.17 µA cm^−2^, and 0.12 µW cm^−2^, compared with 0.56 V, 0.026 µA cm^−2,^ and 0.0039 µW cm^−2^ for the sample with randomly aligned BT nanofibers.^[^ ^19^ ^]^

#### 3.2.8 Electret/Flexoelectric Coupling

Similar to the coupling effect in TENGs, the coupling of the piezoelectric effect with other effects enhances the performance of PENGs. There are two typical approaches: the coupling of the i) electret and ii) flexoelectric effects. The construction of porous dielectric structures is a simple and cost‐effective method of producing electrets. Under the intense polarization‐inducing effect of piezoelectric IDMs, charge separation occurs in the interior of the porous structures, forming pore electrets (Figure 6j). To perfect this polarization‐inducing effect, the IDM must be evenly distributed around the dielectric pores. The most common method involves embedding inorganic piezoelectric micro‐/nanoparticles into a porous polymer matrix.^[^ ^22^ ^]^ Under an external force, the dipole moments of these pore dipoles change in comparison with those of the piezoelectric dipoles, leading to a coupled output. The coupling of the electret effect contributed significantly to the enhancement of the PENG output. For example, Jiang et al. fabricated a hierarchical porous PVDF foam embedded with 30 wt% BT nanoparticles (Figure 6k).^[^ ^107^ ^]^ The corresponding PENG delivered high outputs of 25.66 V and 677.45 nA, which are thirteen times those of the device without the coupling effect.

Many inorganic piezoelectric materials have a high flexoelectric coefficient, making it easy to couple the piezoelectric and flexoelectric effects in specially designed micro‐/nanoarchitectured inorganic piezoelectric materials.^[^ ^13b^ ^]^ Specialized microstructural designs aim to induce a stress gradient in piezoelectric materials under an external force. Piezoelectric and flexoelectric polarizations are generated in response to direct mechanical stress and strain gradients, respectively. Qi et al. reported a PENG comprising buckled PZT ribbons (5−10 µm wide and 250−500 nm thick, Figure 6l), which yielded a strain gradient as high as 3.0 × 10^4^ m^−1^ and a 70% enhancement in piezoelectric effect, with a piezoelectric–flexoelectric coefficient reaching up to 130 pm V^−1^.^[^ ^108^ ^]^ Similarly, Zeng et al. fabricated a PENG consisting of a PVDF‐NNO nanofiber nonwoven fabric sandwiched between two conducting fabric electrodes to achieve piezoelectric–flexoelectric coupling (Figure 6m).^[^ ^109^ ^]^ The coupling effect originates from a non‐uniform deformation field under the interaction between the PVDF‐NNO nanofiber mat and the fabric electrodes. Because of the scaling effect of the gradient‐associated flexoelectricity, its contribution to the coupled output became more pronounced as the material size decreased. It may even surpass the contribution of the piezoelectric effect when the material size is smaller than the micrometer scale.^[^ ^7b^ ^]^

#### 3.2.9 Constructing Multiple Boundary Interfaces

As shown in Equation (12) and (18), the electrode area is strongly related to the output charge and current of a PENG. The electrode serving as boundary interface in the PENG is like a “charge gate,” which manages polarization charge and displacement current inside the device and mobile charge and capacitance conduction current in the external circuit (Figure 2a). Increasing the electrode area in two dimensions can boost the device output charge and current; however, the densities of the output charge and current do not increase. Qin's group developed a 3D intercalation electrode (Figure 6n) to increase the charge and current densities of a PENG.^[^ ^87h^ ^]^ The 3D intercalation electrode with six fins each with an interval of 110 µm created multiple boundary interfaces inside the piezoelectric material, improved the amount of surface polarization charges, and led to 6‐fold enhancement in output charge and current densities. In addition, PVDF‐filled samarium‐doped Pb(Mg_1/3_Nb_2/3_)O_3_‐0.31PbTiO_3_ (Sm‐PMN‐PT) nanowires were selected as piezoelectric materials to further improve the performance. Owing to the superior piezoelectric coefficient of Sm‐PMN‐PT nanowires (142 pm V^−1^) and the nanocomposite structure (Figure 6o), the outputs of the PENG can reach up to 169 nC cm^−2^ and 290 µA cm^−2^.

Briefly, MNIDM‐based PENGs demonstrate outstanding output performances, although they were still lower than those of the MNIDM‐based TENGs. However, unlike TENGs, which require gaps between the functional layers, PENGs offer better structural integrity and easier encapsulation, ensuring excellent working stability and output performance in high‐humidity environments. PENGs designed for biomechanical energy‐to‐electricity conversion are typically worn on the body and are not exposed to extreme conditions, such as high temperatures. Their long‐term stability risks mainly arise from potential structural damage owing to human movement, including the degradation of inorganic microstructures and interface delamination in organic–inorganic composites. With the exception of some PENGs based on high‐quality crystals, PENGs have lower manufacturing costs than TENGs based on similar MNIDMs owing to fewer functional layers.

### 3.3 FENGs Based on MNIDMs

As discussed in Section 2.2, the flexoelectric effect results from a nonuniform strain field, especially the strain gradient.^[^ ^111^, ^112^, ^113^, ^114^, ^115^, ^116^, ^117^ ^]^ Because material strain varies significantly at the micro‐/nanoscale, flexoelectricity exhibits strong size‐dependent effects.^[^ ^9c^ ^]^ In addition, first‐principles calculations revealed that the flexoelectricity is proportional to the permittivity.^[^ ^111^ ^]^ Therefore, MNIDMs with high permittivities, such as nanoshells, nanofibers, and nanosheets based on PZT, BT, and barium strontium titanate (BST), are ideal candidates for realizing large flexoelectric effects.^[^ ^7b^ ^]^ These classic IDMs are also piezoelectrics, enabling the coupling of piezoelectric and flexoelectric effects, and are thus efficient biomechanical‐to‐electrical energy conversion devices.^[^ ^7b^ ^]^ To meet the application requirements of wearable electronics, micro‐/nanoarchitectured inorganic flexoelectric materials should be flexible and easy to mass produce. Meanwhile, although FENGs with uniform functional microstructures can still generate flexoelectric outputs under nonuniform deformation, they lapse under uniform external forces.^[^ ^13b^ ^]^ It is therefore better to design nonuniform microstructures to make FENGs available even under uniform external forces. These types of micro‐/nanoarchitecture designs in wearable FENGs are reviewed in the following sections, focusing on autologous and allosome nonuniform structural designs.

#### 3.3.1 Autologous Nonuniform Structural Design

**Figure 7:**
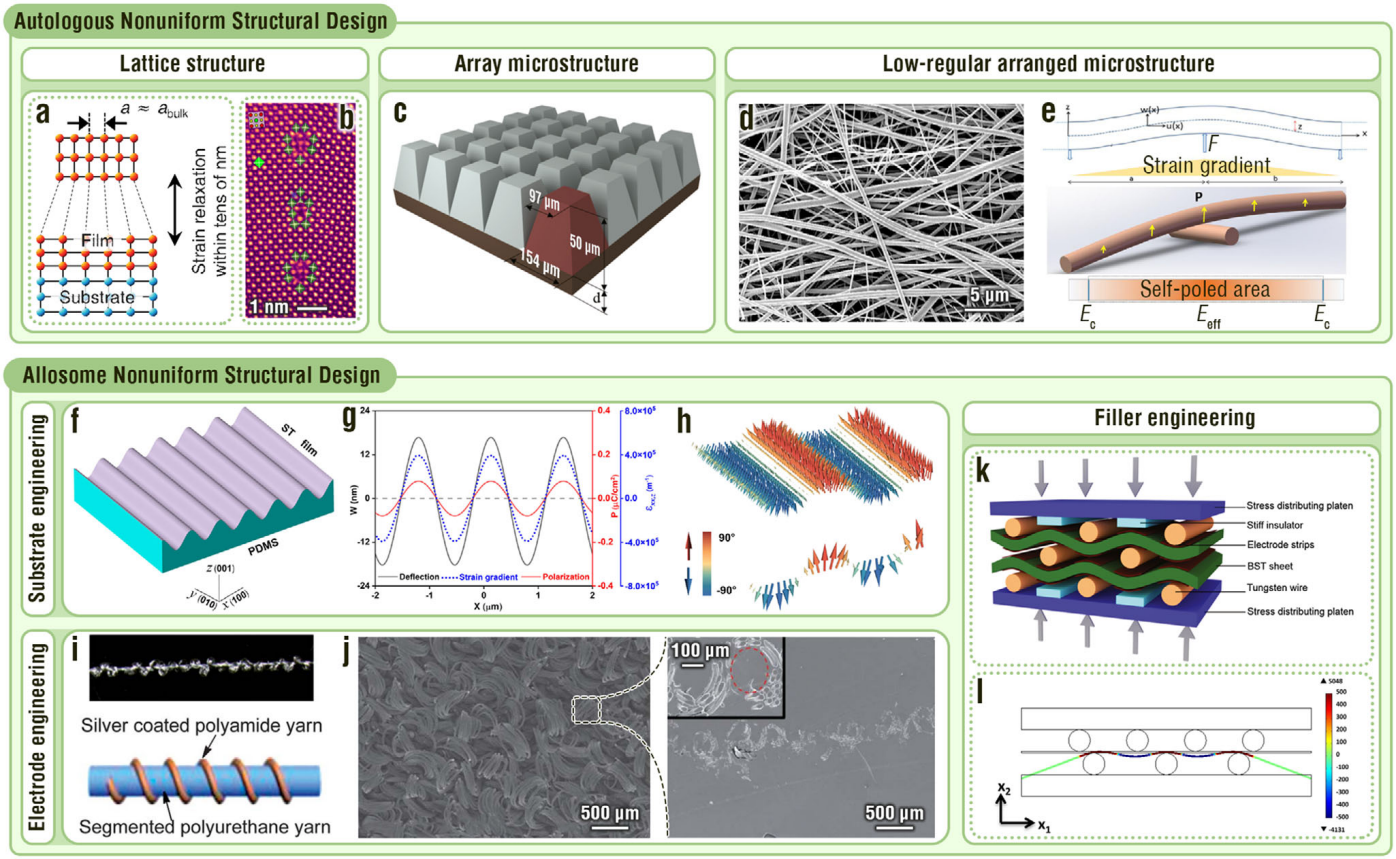
MNIDM‐based FENGs with autologous and allosome nonuniform structural design. a–e) Autologous nonuniform structural design of MNIDM‐based FENGs. a) Schematic diagram showing the lattice mismatch between the epitaxial HoMnO_3_ thin film and the Pt/Al_2_O_3_ substrate. Reproduced with permission.^[^ ^112^ ^]^ Copyright 2011, American Physical Society. b) Polarization at the dislocations in SrTiO_3_. Reproduced with permission.^[^ ^113^ ^]^ Copyright 2018, American Physical Society. c) Schematic diagram of the micropyramid BST array. Reproduced with permission.^[^ ^114^ ^]^ Copyright 2007, American Institute of Physics. d) SEM image of the flexoelectric PZT fiber cluster. e) Schematic diagram showing the strain gradient and upward flexoelectric polarization. Reproduced with permission.^[^ ^28^ ^]^ Copyright 2018, Elsevier. f–l) Allosome nonuniform structural design of MNIDM‐based FENGs. f) Schematic diagram of the ST film transferred on a striated wrinkled PDMS substrate. g) Curves of the deflection, strain gradient, and flexoelectric polarization of the ST film. h) Spatial structure and a section of the wrinkle‐induced flexoelectric polarization. Reproduced with permission.^[^ ^115^ ^]^ Copyright 2024, American Physical Society. i) Schematic structure of the electrode comprising silver‐coated polyamide filament yarns wrapping around segmented polyurethane yarns. j) SEM images of the nonuniformly micro‐nanoarchitectured electrode. Reproduced with permission.^[^ ^109^ ^]^ Copyright 2013, Royal Society of Chemistry. k) 3D view of the FENG fabricated by inserting fine tungsten wires among parallel aligned BST bars. Reproduced with permission.^[^ ^116^ ^]^ Copyright 2009, American Institute of Physics. l) Stimulated strain gradient in the BST bar of the FENG. Reproduced with permission.^[^ ^117^ ^]^ Copyright 2017, Springer.

An autologous nonuniform structure refers to a nonuniform microstructure existing in the flexoelectric material in the absence of an external force. These micro‐/nanoarchitectures primarily include i) inhomogeneous lattice structures, ii) array microstructures, and iii) low‐regular arrangement microstructures. Inhomogeneous lattice structures can be constructed by introducing a lattice mismatch between the epitaxial oxide thin film and substrate^[^ ^112^, ^118^ ^]^ (**Figure** **7**a) or by creating dislocations at the grain boundary (Figure 7b).^[^ ^113^ ^]^ The corresponding device with an inhomogeneous lattice structure inherently exhibited flexoelectric polarization in the absence of an external force. When an external force is applied to the device and the original flexoelectric polarization changes, the FENG can output electrical signals to the external circuit. By contrast, for FENGs with array microstructures, there is generally no flexoelectric polarization in the absence of external forces. When the device is subjected to an external force (even if the force is uniform), a strain gradient and resultant flexoelectric polarization will form in the nonuniform microstructure unit, leading to an electrical output.

Microarrays with gradient geometries are common array microstructures. For example, Fu et al. fabricated micropyramid ceramic arrays with a unit height of 50 µm to create a strong strain gradient in paraelectric phase BST, realizing a high flexoelectric response of 41 pC N^−1^ (Figure 7c).^[^ ^114^ ^]^ A typical example of a low‐regular‐arrangement microstructure is a microfiber cluster.^[^ ^28^, ^119^ ^]^ Zhu et al. observed clear flexoelectric signals from various ceramic microfiber clusters (viz., barium zirconate titanate–barium calcium titanate (BZT‐BCT), potassium sodium antimony‐doped niobate–bismuth sodium potassium zirconate (KNNS‐BNKZ), and PZT fiber clusters) deposited on flat substrates (Figure 7d).^[^ ^28^ ^]^ Because of the autologous confinement of the fibers in space, the upper fibers deposited on the bottom fibers were curved, inducing a strain gradient and upward flexoelectric polarization (Figure 7e). The corresponding PENG delivered a high voltage of up to 9 V.

#### 3.3.2 Allosome Nonuniform Structural Design

In addition to the autologous nonuniform structural design, a strain gradient can also be exerted on flexoelectric materials via an allosome nonuniform microstructural design, i.e., by constructing micro‐/nanostructures on components of the FENG other than the flexoelectric material. Common strategies include the i) substrate, ii) electrode, and iii) filler engineering. To introduce substrate engineering, Shang et al. fabricated a striated wrinkled PDMS substrate and transferred a 20‐nm‐thick 2D paraelectric ST film onto it (Figure 7f).^[^ ^115^ ^]^ The wrinkled substrate rendered the ST film with an enormous periodic strain gradient of 10^5^ m^−1^ (Figure 7g), resulting in periodic flexoelectric polarization as high as 100 nC cm^−2^ (Figure 7g,h). For a flexoelectric material sandwiched between a pair of electrodes, the external force is transmitted to the material through the electrodes. Designing specific micro‐/nanoarchitectured electrodes will facilitate the improvement of the strain gradient. Zeng et al. assembled a nanogenerator with silver‐coated polyamide filament yarns wrapped around segmented polyurethane yarns as the electrodes (Figure 7i) and a PVDF‐NNO nanofiber nonwoven fabric as the flexoelectric material to impose a greater flexoelectric effect.^[^ ^109^ ^]^ The nonuniformly micro‐/nanoarchitectured electrodes (Figure 7j) have a higher Young's modulus (841 MPa) than that of the nanofiber nonwoven fabric (42 MPa). This makes the nonwoven fabric much easier to deform where it is not constrained by adjacent silver‐coated polyamide yarns, inducing a nonuniform local deformation field and thus creating an intensive strain gradient.

Moreover, inserting micro‐/nanoarchitectured fillers into flexoelectric materials is also effective for building and boosting the strain gradient. Chu et al. inserted fine tungsten wires among parallel aligned BST bars (Figure 7k) to generate a strong transverse strain gradient (Figure 7l) in a FENG,^[^ ^117^ ^]^ achieving a giant flexoelectric response (≈3700 pC N^−1^ measured above its Curie temperature) even much higher than those of traditional piezoelectric ceramics.^[^ ^116^ ^]^ It is noteworthy that for FENGs with an allosome nonuniform structural design, the directions of the local strain gradients may not be completely consistent, which will cancel the induced flexoelectric polarization to some extent. Therefore, to avoid flexoelectric polarization cancellation, the electrodes should be arranged to cover regions with signs similar to the strain gradient.

In brief, given the size and permittivity dependence of flexoelectricity, MNIDMs are crucial for enhancing the electrical performance of FENGs, making them comparable to PENGs in terms of output. Unlike most piezoelectrics, flexoelectricity does not rely on non‐centrosymmetric crystal structures, providing a broad range of material options. This opens up the possibility of discovering cost‐effective high‐performance flexoelectric materials. However, current high‐performance FENGs based on MNIDMs depend mainly on costly micro‐/nanofabrication techniques. In addition, their delicate inorganic micro‐/nanostructures pose challenges to their long‐term working stability. Therefore, it is essential to optimize the fabrication cost, robustness, and durability of FENGs for practical applications.

## 4 Applications of MNIDM‐Based Mechanical‐to‐Electrical Energy Conversion Devices

The design of MNIDMs not only endows TENGs, PENGs, and FENGs with high electrical performance but also with light weight, small size, good flexibility, superior robustness, excellent wearability, and even outstanding biocompatibility. These advantages make MNIDM‐based mechanical‐to‐electrical energy‐conversion devices promising candidates for efficient bioenergy harvesting and physiological information sensing. This section discusses the potential applications of TENGs, PENGs, and FENGs with MNIDMs.

### 4.1 Human Biomechanical Energy Harvesting

Humans are rich sources of mechanical energy.^[^ ^120^, ^121^, ^122^, ^123^, ^124^, ^125^, ^126^, ^127^, ^128^, ^129^, ^130^, ^131^, ^132^, ^133^, ^134^, ^135^, ^136^, ^137^, ^138^, ^139^, ^140^, ^141^ ^]^ An average‐sized person yields as much mechanical energy as the energy of a 250 kg battery, and the biomechanical output power can easily be maintained at over 100 W.^[^ ^124^ ^]^ Traditional biomechanical energy harvesters, such as hand‐crank generators, require the user to focus on power generation at the expense of other activities, causing short bouts of generation. In contrast, TENGs, PENGs, and FENGs can generate electrical power for longer durations. When these NGs are fixed to body parts with joint movement in vitro, or muscles in vivo, multiscale biomechanical energy (from as small as arterial pulsation to as large as running) is constantly harvested during daily activities. The harvested energy can be stored in capacitors and batteries or can directly drive small electronics in high‐entropy energy supply networks, human–machine interaction systems, and the IoT.

#### 4.1.1 In Vitro Biomechanical Energy Harvesting

**Figure 8:**
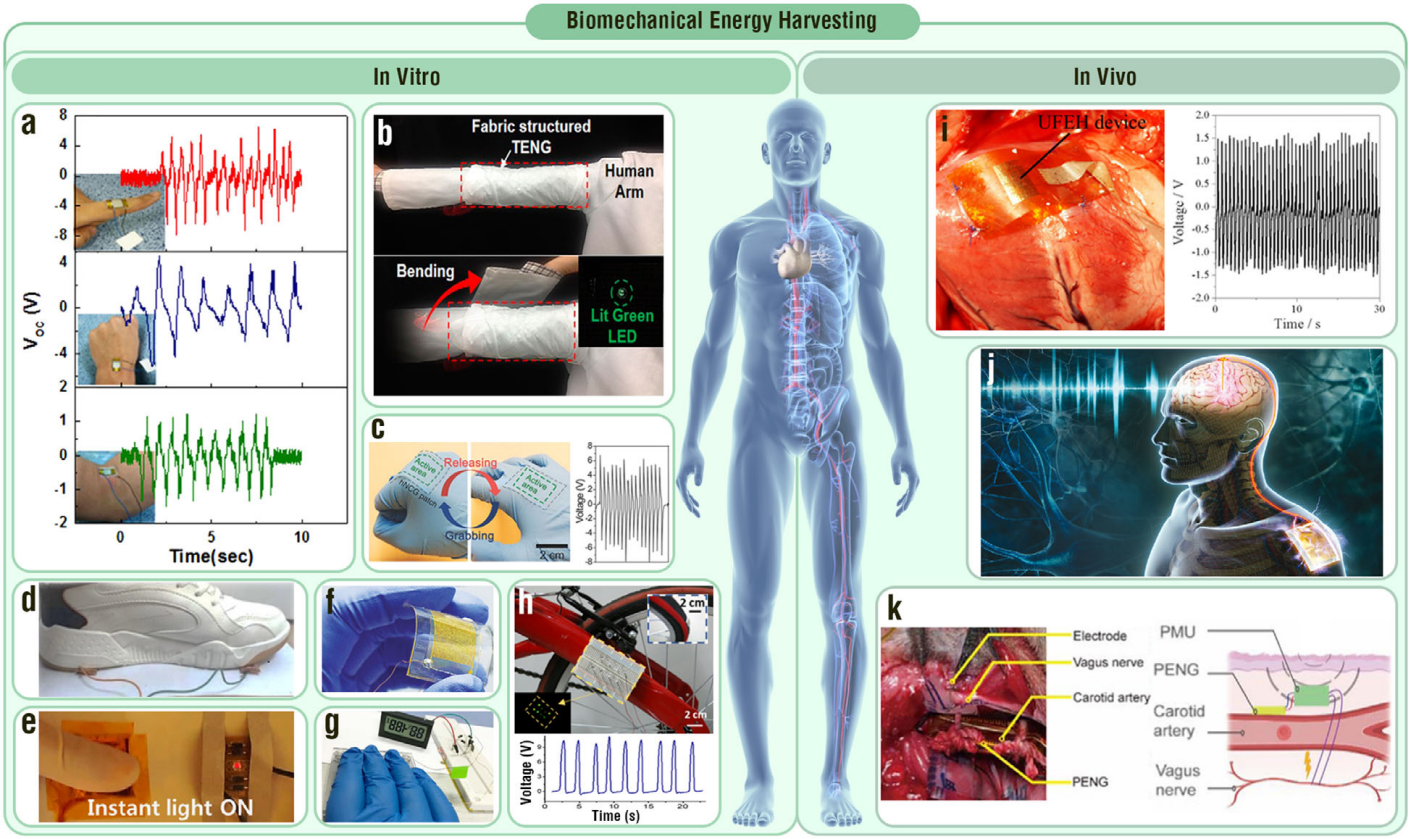
Applications of MNIDM‐based mechanical‐to‐electrical energy conversion devices in biomechanical energy harvesting. a) Energy harvesting results of BT nanoparticle‐based PENGs respectively attached to index finger, wrist, and elbow. Reproduced with permission.^[^ ^87c^ ^]^ Copyright 2015, Elsevier. b) Lighted light‐emitting diode (LED) via a ZnO nanowire‐based TENG attached to an arm. Reproduced with permission.^[^ ^70d^ ^]^ Copyright 2015, American Physical Society. c) Energy harvesting via a BT nanowire‐based PENG attached to the back of a hand. Reproduced with permission.^[^ ^125^ ^]^ Copyright 2018, Wiley‐VCH. d) Mica‐based TENG shoe. Reproduced with permission.^[^ ^70b^ ^]^ Copyright 2021, American Physical Society. e) Lighted LED via a hemispherically aggregated BT nanoparticle‐based PENG upon a finger impact. Reproduced with permission.^[^ ^23^ ^]^ Copyright 2014, American Physical Society. f) Energy harvesting by bending a PZT thin‐film‐based PENG. Reproduced with permission.^[^ ^126^ ^]^ Copyright 2014, Royal Society of Chemistry. g) Energy harvesting by patting a PLZT thin‐film‐based TENG. Reproduced with permission.^[^ ^9b^ ^]^ Copyright 2021, Elsevier. h) Lighted LEDs via a BT nanoparticle‐based PENG fixed on the bicycle. Reproduced with permission.^[^ ^22^ ^]^ Copyright 2018, American Physical Society. i) Implantable PZT thin‐film‐based PENG for powering a pacemaker. Reproduced with permission.^[^ ^127^ ^]^ Copyright 2015, Springer Nature. j) Implantable PIMNT thin‐film‐based PENG for deep brain stimulation. Reproduced with permission.^[^ ^128^ ^]^ Copyright 2015, Royal Society of Chemistry. k) Implantable BT nanoparticle‐based PENG for vagal neuromodulation. Reproduced with permission.^[^ ^129^ ^]^ Copyright 2021, Elsevier.

**Figure 9:**
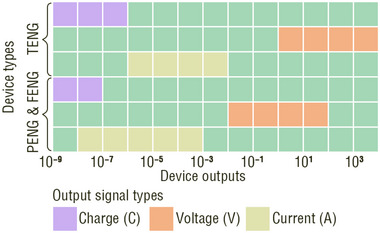
Comparison of general output electrical performance for MNIDM‐based TENGs, PENGs, and FENGs.

Human biomechanical energy is derived mainly from body movements. In vitro MNIDM‐based TENGs, PENGs, and FENGs are promising high‐entropy micropower supplies for efficiently converting mechanical energy into electricity to power electronics in the IoT. These devices are generally attached to the body parts that involve joint movements to generate superior equivalent electric power. Common body parts for mounting these devices include the fingers, wrists, elbow (**Figure** **8**a), arm (Figure 8b), back of the hand (Figure 8c), and feet.^[^ ^70^, ^87^, ^125^ ^]^ For instance, the use of TENGs based on SiO_2_ micro‐nanoparticles in wearable devices to harvest energy from human motion has been demonstrated by Lv's group.^[^ ^130^ ^]^ Moreover, integrating these NGs into facilities with intensive human activity is another effective approach for harvesting biomechanical energy. When a person directly steps on (Figure 8d), presses (Figure 8e), pinches (Figure 8f), and pats (Figure 8g) these NGs, or indirectly drives the NGs upon operating facilities (e.g., riding a bicycle, Figure 8h), electricity is generated.^[^ ^9^, ^22^, ^23^, ^70^, ^126^ ^]^ For example, the integration of ceramic‐based PENGs in smart flooring systems, which convert foot traffic into electrical energy, was illustrated by Liu's group.^[^ ^131^ ^]^ The harvested electrical energy increases with an increase in both the body movement frequency and force acting on the devices. Furthermore, the triboelectric device has higher output voltage (from several to a few thousand V), current (from several to a few thousand µA), and transferred charge density (from several to several hundred nC) than those of piezoelectric and flexoelectric devices (**Figure** **9**). The output voltage, current, and transferred charge density of piezoelectric and flexoelectric devices are generally in the range of dozens of mV to dozens of V, dozens of nA to several hundred µA, and several to several dozen nC, respectively. Nonetheless, piezoelectric and flexoelectric devices possess higher output stabilities in high‐humidity environments than triboelectric devices because moisture can rapidly dissipate the triboelectric surface charge.^[^ ^4b^ ^]^ Therefore, it is necessary to select appropriate biomechanical energy harvesters based on the practical application scenarios.

#### 4.1.2 In Vivo Biomechanical Energy Harvesting

Achieving an uninterrupted and reliable power source for implantable biomedical devices is challenging. The bulkiness, limited capacity, and short shelf life of currently used batteries pose safety hazards, surgical risks, and expensive surgical procedures for battery recharge/replacement.^[^ ^132^ ^]^ MNIDM‐based NGs featuring low weight, low thickness, and powerful output are ideal candidates for extending the working life and reducing the risks of implantable biomedical devices. Furthermore, the output electrical pulses of in vivo MNIDM‐based NGs can be used directly to treat diseases. For example, Lu et al. reported an ultrathin ultra‐flexible PZT thin‐film‐based PENG to harvest the energy of the heartbeat to power a pacemaker (Figure 8i).^[^ ^127^ ^]^ The ultra‐thin ultra‐flexible PENG was fixed from the left ventricular apex to the right ventricle without any burden or damage to the heart and delivered a peak‐to‐peak voltage as high as 3 V. Hwang et al. developed an implantable Pb(In_1/2_Nb_1/2_)O_3_–Pb(Mg_1/3_Nb_2/3_)O_3_–PbTiO_3_ (PIMNT) thin‐film‐based PENG, which served as a self‐powered deep brain stimulation device (Figure 8j).^[^ ^128^ ^]^ A PIMNT thin‐film‐based PENG can convert tiny bodily mechanical motions into electricity. With a slight strain triggering, an ultrahigh current (0.57 mA) was generated over the threshold for real‐time deep brain stimulation. Recently, Zhang et al. reported a self‐powered vagus nerve stimulation device based on a PENG comprising BT nanoparticle–PVDF‐TrFE composite nanofibers.^[^ ^129^ ^]^ This in vivo device harvests biomechanical energy from carotid artery pulsation to stimulate the vagus nerve for various inflammatory, immune, neuropsychiatric, and cardiovascular disease treatments (Figure 8k).

### 4.2 Human Physiological Information Sensing

The high electrical performance of MNIDM‐based NGs results in a high signal‐to‐noise ratio, low detection limit, fast response, and high linearity, making them suitable for sensing human physiological information. In the following section, we focus on the applications of MNIDM‐based NGs in various physiological information detection, including i) human body movement monitoring, ii) pulse monitoring, iii) blood pressure monitoring, iv) heart monitoring, and v) sound sensing.

#### 4.2.1 Human Body Movement Monitoring

**Figure 10:**
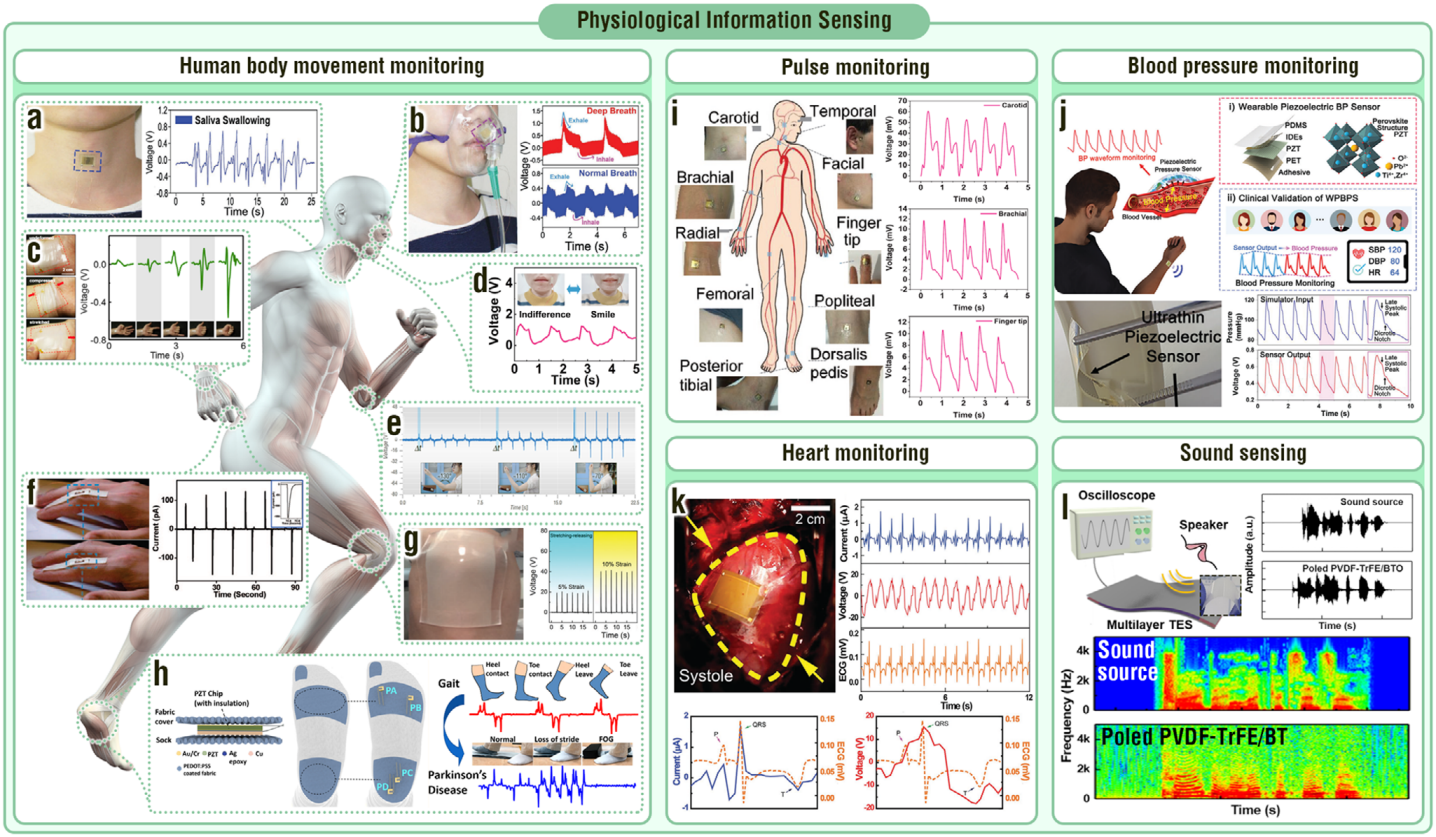
Applications of MNIDM‐based mechanical‐to‐electrical energy conversion devices in physiological information sensing. a) PZT thin‐film‐based PENG attached to the middle of the throat for saliva swallowing action monitoring. b) PZT thin‐film‐based PENG attached to the medical mask for human respiration monitoring. Reproduced with permission.^[^ ^133^ ^]^ Copyright 2017, Wiley‐VCH. c) ZnO nanowire‐based PENG mounted on the wrist for human gesture monitoring. Reproduced with permission.^[^ ^53a^ ^]^ Copyright 2014, American Chemical Society. d) ZnO nanoparticle‐based PENG attached to the face for human expression monitoring. Reproduced with permission.^[^ ^134^ ^]^ Copyright 2023, Springer Nature. e) PLZT thin‐film‐based TENG attached to the elbow for arm motion monitoring. Reproduced with permission.^[^ ^9b^ ^]^ Copyright 2021, Elsevier. f) ZnO nanowire‐based PENG attached to the index finger for finger action monitoring. Reproduced with permission.^[^ ^85^ ^]^ Copyright 2015, Elsevier. g) PZT microfoam‐based PENG attached to the knee for knee motion monitoring. Reproduced with permission.^[^ ^15a^ ^]^ Copyright 2018, Royal Society of Chemistry. h) PZT‐based PENGs integrated into the sock for sports monitoring. Reproduced with permission.^[^ ^139^ ^]^ Copyright 2019, American Chemical Society. i) III‐N thin‐film‐based PENG for pulse monitoring. Reproduced with permission.^[^ ^133^ ^]^ Copyright 2019, Wiley‐VCH. j) PZT thin‐film‐based PENG for blood pressure monitoring. Reproduced with permission.^[^ ^138^ ^]^ Copyright 2023, Wiley‐VCH. k) Mn doping PMN‐PZT‐Mn thin‐film‐based PENG for heart monitoring. Reproduced with permission.^[^ ^140^ ^]^ Copyright 2017, Wiley‐VCH. l) BT nanoparticle‐based TENG for sound sensing. Reproduced with permission.^[^ ^24^ ^]^ Copyright 2020, American Chemical Society.

MNIDM‐based NGs have shown significant potential for human body movement monitoring in healthcare and kinematic analysis. These NGs can be attached to body parts with joint or muscle movements for a long time to detect actions or postural information in real‐time. For example, it has been demonstrated that saliva‐swallowing action (**Figure** **10**a), respiration (Figure 10b),^[^ ^133^ ^]^ gesturing (Figure 10c),^[^ ^53a^ ^]^ facial expression (Figure 10d),^[^ ^134^ ^]^ arm motion (Figure 10e),^[^ ^9b^ ^]^ finger action (Figure 10f),^[^ ^85^ ^]^ knee motion (Figure 10g),^[^ ^15a^ ^]^ and gait (Figure 10h)^[^ ^135^ ^]^ can be precisely monitored via MNIDM‐based NGs. In terms of healthcare, these body movement signals contribute to the early detection and prevention of certain health issues. For example, abnormal incipient patterns in Parkinson's disease and musculoskeletal disorders can be detected by hand and leg movements and gait.^[^ ^135^ ^]^ The real‐time monitoring of human posture helps correct an individual's unhealthy posture and prevents scoliosis, kyphosis, cervical spondylosis, and lumbar disc herniation.^[^ ^136^ ^]^ In addition, NG‐based self‐powered body‐movement monitoring systems combined with the IoT and remote monitoring technologies provide unprecedented possibilities for rehabilitation and physical therapy.^[^ ^137^ ^]^ By analyzing movements, healthcare providers can develop personalized rehabilitation programs that target specific deficits in mobility or strength. In terms of kinematic analysis, comprehensive body movement information obtained by MNIDM‐based NGs can help athletes and coaches enhance their performance by fine‐tuning movement patterns, such as running or swimming stroke techniques, based on detailed biomechanical feedback.^[^ ^9b^ ^]^ Moreover, monitoring movements can identify potentially harmful techniques or imbalances that predispose athletes to injuries. These data are crucial for developing training regimens to minimize the risk of injury.

#### 4.2.2 Pulse Monitoring

Pulse signals are important indicators of an individual's physiological and psychological health.^[^ ^1a^ ^]^ MNIDM‐based NGs with low detection limits, fast responses, thin structures, and good flexibility are suitable for obtaining precise pulse signals with excellent comfort. These NGs can detect pulse signals when attached to the epidermis covering the arteries, such as the carotid, brachial, radial, femoral, posterior tibial, dorsalis pedis, femoral, popliteal, fingertip, facial, and temporal artery signals (Figure 10i).^[^ ^133^ ^]^ Pulse rate and pulse rate variability are the two most readily available indicators^[^ ^138^ ^]^ that reflect physical activity level, cardiac function, mental stress, and emotional information.^[^ ^1a^ ^]^ More indistinguishable pulse signals of the systolic peak (P_1_), reflected systolic peak (P_2_), and dicrotic notch (P_3_) can be detected using highly sensitive devices, thereby expanding the accessible health information. Recently, a III‐N thin‐film‐based PENG system was developed to monitor fine pulse waves comprising P_1_, P_2_, and P_3_ in various body parts (Figure 10i). Two important indicators, the augmentation index (AIx) and pulse wave velocity (PWV), obtained through the ratio of P_1_ to P_2_ and the time delay between two pulse sites, respectively, can help estimate blood vessel hardness and cardiovascular risk.

#### 4.2.3 Blood Pressure Monitoring

Blood pressure monitoring is important for the early warning of hypertension and the prevention of stroke, cardiac failure, myocardial infarction, renal failure, dementia, blindness, and even sudden death.^[^ ^141^ ^]^ Blood pressure information can also be acquired using MNIDM‐based NGs, which achieve portable, wearable, and continuous signal monitoring technology compared with traditional cuff‐based sphygmomanometers.^[^ ^141^ ^]^ The NG can be directly fixed around the artery or body surface close to the arteries for in vivo and in vitro blood pressure monitoring, respectively.^[^ ^132^, ^142^ ^]^ To obtain the actual blood pressure signals, the electrical signals recorded from NGs must be corrected by mathematical conversion and calibration.^[^ ^142^ ^]^ Therefore, it is necessary to design devices with high sensitivity and linearity. Recently, Min et al. developed a PZT thin‐film‐based PENG that exhibit a wide linear pressure sensing range of 0–10 kPa and a high sensitivity of 0.062 kPa^−1^ (Figure 10j).^[^ ^138^ ^]^ Because the pressure exerted by the pulse waves in the artery fall within 10 kPa, a simple linear regression approach was used to determine the blood pressure. The measurement accuracy of the PENG and the transfer function were demonstrated by calculating the mean difference between the results of the device and those of a commercial sphygmomanometer. The mean difference between them was only −0.89 ± 6.19 and −0.32 ± 5.28 mmHg for systolic blood pressure and diastolic blood pressure, respectively.

#### 4.2.4 Heart Monitoring

Early detection of abnormalities or variations in cardiac cycle functionality plays a crucial role in preventing heart diseases and related complications. MNIDM‐based NGs can be directly fixed to the heart to replace multiwire electrocardiograph (ECG) systems for long‐duration heart monitoring, such as heart rhythm and rate. This is because heart activities simultaneously generate electrical and mechanical vibration signals that correspond to each other, which enables the time‐domain voltage signal of the NG to be mapped to an equivalent ECG time‐domain trace.^[^ ^143^ ^]^ The mapping relationship between the two can be clarified through signal processing techniques, such as the convolution theorem and Fourier transform. Kim et al. prepared a 0.5 mol% Mn doped 0.4Pb(Mg_1/3_Nb_2/3_)O_3_−0.6Pb(Zr,Ti)O_3_ (PMN‐PZT‐Mn) thin‐film‐based PENG and fixed it on the epicardium for heart monitoring.^[^ ^140^ ^]^ The characteristic current and voltage signals generated from the PENG accurately coincided with the ECG signals from the heart. Three key characteristic waves, the P wave (corresponding to atrial depolarization), QRS wave (corresponding to ventricular depolarization), and T wave (corresponding to the repolarization of the ventricles), were synchronized between the piezoelectric and ECG signals (Figure 10k). This indicates that the electrical response of MNIDM‐based NGs can be directly mapped to the cardiac cycle physiology. In addition, Li's group developed a TENG capsule based on glass micropellets wrapped in PTFE powder, which served as a zero‐power consumption and implantable heart monitor to detect abnormal changes in cardiac contractility.^[^ ^144^ ^]^

#### 4.2.5 Sound Sensing

Owing to their fast response and high sensitivity, MNIDM‐based NGs can also serve as sound sensors, exhibiting the potential for use in self‐powered microphones, voice security systems, and healthcare. Park et al. reported a self‐powered TENG microphone based on a multilayered PVDF‐TrFE/BT nanoparticle composite film.^[^ ^24^ ^]^ This device can record acoustic waves within a frequency range of 0.1–8 kHz. The recorded acoustic waves and short‐time Fourier transform signals of the multilayered MNIDM‐based sensor matched well with those of the original source (Figure 10l). In addition to recording human voices, acoustic NGs can be used to detect heart, Korotkoff, and breath sounds for health monitoring; to acquire these sounds, NG sensors can be attached to the left chest, upper arm, and lungs or trachea region, respectively. The heart, Korotkoff, and breath sound information detected by the NGs can help diagnose heart valve function, heart disease, respiratory disease, and other conditions in a timely and real‐time manner.^[^ ^145^ ^]^

## 5 Summary and Outlook

**Figure 11:**
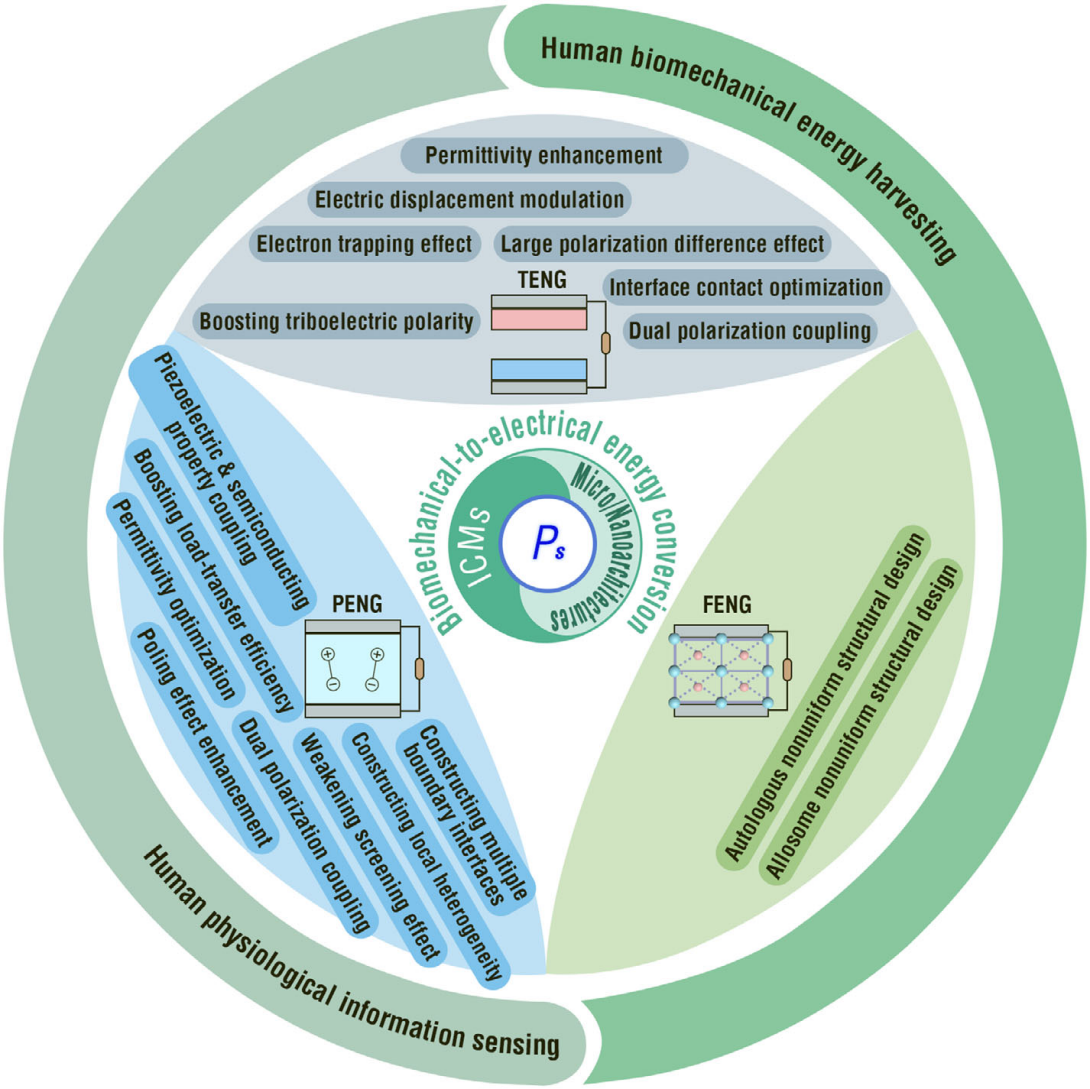
Summary diagram of theoretical framework, design strategies, and application demonstration of biomechanical‐to‐electrical energy conversion devices based on MNIDMs.

In this review, we first interpreted the working mechanisms, equivalent capacitor models, operation modes, and output characteristics of TENGs, PENGs, and FENGs as advanced biomechanical‐to‐electrical energy conversion devices. We then elaborated on the unique advantages of designing various IDMs coupling micro‐/nanoarchitectures to enhance the electrical performance of TENGs, PENGs, and FENGs. Finally, we reviewed the potential applications of MNIDM‐based TENGs, PENGs, and FENGs for bioenergy harvesting and physiological information sensing (**Figure** **11**).

**Figure 12:**
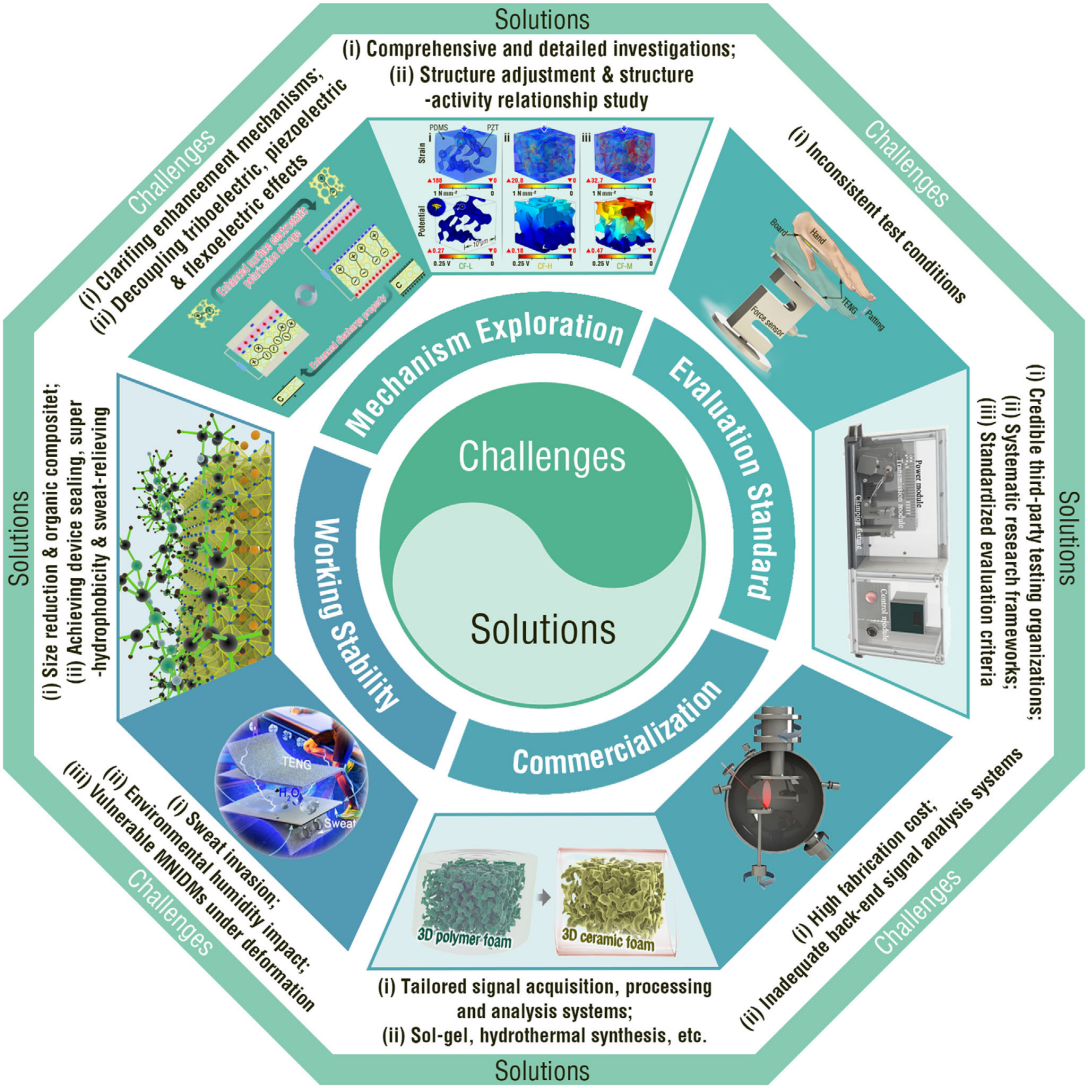
Challenges and corresponding potential solutions in the development of biomechanical‐to‐electrical energy conversion devices based on MNIDMs.

It has been demonstrated that MNIDMs endow TENGs, PENGs and FENGs with unparalleled usability and excellent ability to convert human mechanical signals into electrical signals for bioenergy scavenging and personal healthcare monitoring. Compared with NGs based on organic materials or lacking micro‐/nanoarchitectures, MNIDM‐based devices usually exhibit superior electrical output performances and sensing signal‐to‐noise ratios owing to the excellent polarization regulation capability of MNIDMs.^[^ ^8^, ^20^ ^]^ Furthermore, MNIDM‐based TENGs, PENGs, and FENGs, with their dainty structures, good flexibility, and outstanding electrical performance, have gained an edge in convenience, comfort, real‐time monitoring, and working continuity in contrast to traditional electromagnetic biomechanical energy generators and commercial medical monitoring devices.^[^ ^4^, ^9^, ^76^ ^]^ These MNIDM‐based devices are promising for powering small electronics in the IoT and for sensing human body movements, pulse, blood pressure, cardiac activity, and sound.^[^ ^133^, ^138^, ^140^ ^]^ Despite the aforementioned advancements in biomechanical‐to‐electrical energy conversion devices based on MNIDMs, various practical challenges remain. We outline some of these challenges and propose relevant solutions below (**Figure** **12**).

### 5.1 Mechanism Exploration

The future evolution of MNIDM‐based biomechanical‐to‐electrical energy conversion devices requires the clarification of their performance enhancement mechanisms and decoupled triboelectric, piezoelectric, and flexoelectric effects on the hybrid electrical output. Despite various hypotheses regarding how MNIDMs enhance the electrical performance of NGs, the underlying mechanisms remain uncertain. For example, embedding inorganic piezoelectric micro‐/nanoparticles into organic tribo‐material matrices boosts the electrical outputs.^[^ ^59^ ^]^ Whether this enhancement stems from triboelectric–piezoelectric polarization coupling or permittivity enhancement is unclear. If the piezoelectric polarizations in the domains cancel each other owing to the random distribution of piezoelectric particles, the permittivity enhancement dominates. In contrast, if the triboelectric surface charge‐induced electric field aligns with the polarization of the piezoelectric domains, both polarization coupling and permittivity enhancement contribute to performance gains. Moreover, although effective methods to distinguish the triboelectric and piezoelectric components in hybrid outputs exist,^[^ ^146^ ^]^ isolating the flexoelectric contribution in MNIDM‐based tribo‐/piezoelectric–flexoelectric hybrid NGs remains a challenge. Precise adjustment of the phase structures and micro‐/nanostructures of IDMs, along with systematic studies of the structure–performance relationships, will be key to decoupling the triboelectric, piezoelectric, and flexoelectric effects in a single NG. These efforts will establish a theoretical foundation for the further optimization of device output.

### 5.2 Evaluation Standard

A striking reality of MNIDM‐based biomechanical‐to‐electrical energy conversion devices is that their electrical output performance cannot be easily compared, owing to underperforming test equipment and inconsistent test conditions. For example, MNIDM‐based NGs have ultrashort response times; however, the low sampling rates in some test equipment may lead to overestimated values. Key metrics such as energy conversion efficiency, output voltage, current density, transferred charge density, power density, sensitivity, and response time have been established to evaluate device performance. However, discrepancies in testing situations, such as operating frequency, applied load, temperature, humidity, data analysis approach, calculation methodology, and testing mechanism, hinder direct comparisons across studies. The haphazard state of NG research has hampered clear guidance for future research and for product designers. Therefore, systematic research frameworks, standardized evaluation criteria, and credible third‐party testing organizations should be established as soon as possible to guide the optimization of state‐of‐the‐art biomechanical‐to‐electrical energy conversion devices.

### 5.3 Working Stability

Frequent and intense human mechanical activity creates challenges for the stability of wearable and implantable MNIDM‐based NGs. Compared with organic materials, MNIDMs with markedly higher Young's moduli are more susceptible to damage during long‐term bending, twisting, curling, and folding of devices.^[^ ^15a^ ^]^ With the damage and disappearance of the micro‐/nanoarchitectures of IDMs, the device performance suddenly decreases. The appropriate size reduction of IDMs and their combination with organic materials are ideal approaches for increasing the mechanical robustness and stability of devices.^[^ ^76^ ^]^ This is because, to some extent, smaller IDM sizes offer greater flexibility, and organic materials reinforce MNIDMs while cushioning mechanical impacts.^[^ ^12^, ^18^, ^58^ ^]^ Moreover, external environmental factors such as temperature and humidity changes also affect working stability. Because these devices are body‐mounted, their temperature approximates body temperature; however, humidity exerts a more significant influence on the working stability, especially for TENGs. High humidity promotes the formation of a water layer on functional interfaces, dissipating electrostatic charges and degrading the output. Similarly, sweat invasion also compromises stability. Therefore, it is necessary to seal devices, construct superhydrophobic functional interfaces, and design sweat‐relieving microstructures.

### 5.4 Commercialization

MNIDM‐based TENGs, PENGs, and FENGs have demonstrated potential benefits in real‐time, long‐term, and continuous human biomechanical energy harvesting and physiological information sensing. However, the high fabrication costs of MNIDMs and inadequate signal acquisition, processing, and analysis systems hinder their commercialization. Some MNIDMs are challenging to mass produce or require costly fabrication equipment such as magnetron sputtering, pulsed laser deposition, molecular beam epitaxy, and chemical vapor deposition systems.^[^ ^147^ ^]^ In addition, static force measurement with NGs requires high‐resistance electrometers, which are difficult to miniaturize and prone to baseline drift,^[^ ^1a^ ^]^ making it difficult to monitor static forces in a portable and precise manner. However, they generate high noise, which requires filtering circuits to detect weak physiological signals. Moreover, the existing commercial signal analysis strategies for individual monitoring were originally designed for conventional electrocardiogram, electromyographic, capacitive, and photoelectric signals, making them incompatible with triboelectric, piezoelectric, and flexoelectric signals. Therefore, low‐cost large‐scale manufacturing technologies must be explored. For example, the sol–gel method can serve as a viable alternative to the abovementioned expensive techniques to some extent, enabling large‐scale and low‐cost production.^[^ ^9b^ ^]^ This should be further optimized to produce low‐defect and high‐quality MNIDM crystals, including self‐supported and single crystals. Hydrothermal synthesis, electrospinning, and electrostatic atomization have emerged as cost‐effective and scalable approaches to produce various MNIDMs.^[^ ^4^, ^64^ ^]^ Very recently, a sacrificial template‐sol–gel combination method was developed to fabricate triboelectric and piezoelectric MNIDMs with low cost, high yield, and excellent structural designability.^[^ ^76^ ^]^ Meanwhile, developing tailored signal acquisition, processing and analysis systems for emerging MNIDM biomechanical‐to‐electrical energy conversion devices is indispensable.

Despite these unresolved challenges, we believe that MNIDM‐based TENGs, PENGs, and FENGs will realize their full potential as all of the above aspects continue to evolve. Next‐generation human biomechanical‐to‐electrical energy conversion devices aim to achieve higher electrical performance, better working stability, and easier and cheaper large‐scale production. With advanced energy management, data communication, and analysis technologies, these devices are expected to show practicality not only in the construction of high‐entropy micro‐power supply networks but also in the real‐time long‐term monitoring of personal physiological and psychological information and working and living conditions without affecting the daily activities of the individual. Even in geographically remote or economically limited areas, these devices are poised to work reliably and consistently at the IoT margin, providing comfortable, timely, personalized healthcare, convenient human–computer interaction, and intelligent cloud steward services.

## Conflict of Interest

The authors declare no conflict of interest.

## Author Contributions

J.H.Z. and Z.Li conceived the review. J.H.Z. drafted the review and prepared graphics. Z.Li, X.X., X.H., Y.Y., Y.L., and Z.Liu supervised the review. Y.Y. and X.H. reviewed and refined the review for scientific validity and completeness. N.S., S.Z., C.C. and J.D. contributed to manuscript structure design. M.L. and J.G. organized the references. All authors have read and agreed to the published version of the paper.

## Supporting information



Supporting Information
