# Supplementary material for: Inorganic Dielectric Materials Coupling Micro‐/Nanoarchitectures for State‐of‐the‐Art Biomechanical‐to‐Electrical Energy Conversion Devices
Source: Adv Mater. 2025 May 3;37(28):2419081. doi: 10.1002/adma.202419081 (PMC12272043; doi:10.1002/adma.202419081)
Supplement: Supplementary file 1 — Supporting Information [file ADMA-37-2419081-s001.pdf]

ADVANCED MATERIALS

Supporting Information

for *Adv. Mater.*, DOI 10.1002/adma.202419081

Inorganic Dielectric Materials Coupling Micro-/Nanoarchitectures for State-of-the-Art Biomechanical-to-Electrical Energy Conversion Devices

Jia-Han Zhang, Zhengtong Li, Zeng Liu, Mingxuan Li, Jiaxin Guo, Jinhua Du, Changkun Cai, Shaohui Zhang, Ningning Sun, Yong Li, Xingtao Xu*, Xihong Hao* and Yusuke Yamauchi**

Supporting Information

Inorganic Dielectric Materials Coupling Micro/Nanoarchitectures for State-of-the-Art Biomechanical-to-Electrical Energy Conversion Devices

Jia-Han Zhang, Zhengtong Li, Zeng Liu, Mingxuan Li, Jiaxin Guo, Jinhua Du, Changkun Cai, Shaohui Zhang, Ningning Sun, Yong Li, Xingtao Xu,* Xihong Hao,* and Yusuke Yamauchi**

J.-H. Zhang, Z. Liu, M. Li, J. Guo
Electronic-Photonic Smart Sensing Device R&D Team, Inner Mongolia Key Laboratory of Intelligent Communication and Sensing and Signal Processing, School of Electronic Information Engineering, Inner Mongolia University, Hohhot 010021, China

Z. Li
Key Laboratory of Hydrology Water Resources and Hydraulic Engineering, Hohai University, Nanjing 210098, China
E-mail: 200201030001@hhu.edu.cn

J. Du
School of Chemistry and Chemical Engineering, Inner Mongolia University of Science and Technology, Baotou 014010, China

C. Cai, N. Sun, Y. Li, X. Hao
School of Materials and Metallurgy, Inner Mongolia University of Science and Technology, Baotou 014010, China
E-mail: xihonghao2022@163.com

S. Zhang
National Engineering Research Center for Healthcare Devices & Guangdong Provincial Key

Laboratory of Medical Electronic Instruments and Materials, Institute of Biological and Medical Engineering, Guangdong Academy of Sciences, Guangzhou 510316, China

X. Xu
China Marine Science and Technology College, Zhejiang Ocean University, Zhoushan 316022, China
E-mail: xu.xingtao@nims.go.jp

Y. Yamauchi
Department of Materials Process Engineering, Graduate School of Engineering, Nagoya University, Nagoya 464-8603, Japan
E-mail: y.yamauchi@uq.edu.au

Table S1. Comparison of material compatibilities and scalabilities among MNIDM-based TENGs, PENGs, and FENGs.

<table>
<tr><th>NG Types</th><th>Key material types</th><th colspan="2">Material compatibilities</th><th colspan="2">Scalabilities</th><th>Ref.</th></tr>
<tr><td rowspan="5">TENG</td><td>All-inorganic dielectric materials (e.g., films, nanowire arrays, etc.)</td><td>Stable structure, resistance to aging and high temperatures, but high rigidity leading to the risk of brittleness.</td><td rowspan="7">Most IDMs are non-toxic, while a small fraction with high performance (e.g., the lead-based ceramic) is mildly toxic and requires encapsulation to avoid biocompatibility risks.</td><td>Excellent storage stability, but relatively high synthesis complexity and difficulty in large-scale production.</td><td rowspan="5">With high energy conversion efficiency, MNIDM-based TENGs excel in biomechanical energy harvesting, but they are sensitive to humidity and require encapsulation to ensure stability.</td><td>[1]</td></tr>
<tr><td>IDM skeleton embedded in a polymer matrix</td><td rowspan="2">Improvement in flexibility to some extent, but modulus mismatch leading to interface rupture or delamination.</td><td rowspan="4">Good durability and robustness, can be synthesized via sol-gel, hydrothermal, etc., making large-scale production easier.</td><td>[2]</td></tr>
<tr><td>IDM interlayer</td><td>[3]</td></tr>
<tr><td>IDM particle-polymer composite interlayer</td><td rowspan="2">Good flexibility, but agglomeration risk at high IDM content.</td><td>[4]</td></tr>
<tr><td>IDM particles embedded in a polymer matrix</td><td>[5]</td></tr>
<tr><td rowspan="2">PENG</td><td>Non-centrosymmetric IDMs</td><td rowspan="2">Centrosymmetric (e.g., CGO) and ferroelectric IDMs (e.g., BT) require post poling to activate piezoelectricity, while other piezoelectric materials (e.g., ZnO) do not. Compared to all-inorganic materials, organic-inorganic composites have better flexibility and are less prone to damage during human activities.</td><td colspan="2" rowspan="2">In general, all-inorganic piezoelectrics offer better performance, while organic-inorganic composite piezoelectrics are easier to produce on a large scale. Non-centrosymmetric piezoelectrics have made significant progress in biomechanical-to-electrical energy conversion, while centrosymmetric piezoelectrics are in the early stages of research, and their potential for application requires further investigation.</td><td>[6]</td></tr>
<tr><td>Centrosymmetric IDMs</td><td>[7]</td></tr>
<tr><td rowspan="2">FENG</td><td>Autologous nonuniform structures</td><td colspan="2" rowspan="2">Not limited to non-centrosymmetric crystal structures, the strain gradient can induce the flexoelectricity of IDMs, offering a wide selection of materials and good material compatibility.</td><td colspan="2" rowspan="2">Flexoelectricity possesses significant size dependency. Their reliance on precise micro/nanofabrication limits manufacturing scalability. In contrast to the autologous nonuniform structures, the allosome nonuniform structures, free from further micro/nanofabrication in flexoelectric IDMs, offer more practical potential.</td><td>[8]</td></tr>
<tr><td>Allosome nonuniform structures</td><td>[9]</td></tr>
</table>

References

[1] R. Zhang, M. Hummelgård, J. Örtegren, M. Olsen, H. Andersson, Y. Yang, Z.-L. Wang, H. Olin, P. Sutar, D. Mihailovic, *Nano Energy* **2021**, 89, 106363.

[2] J.-H. Zhang, Z. Li, B. Shen, Z. Liu, L. Chen, H. Wang, H. Li, Y. Zhang, S. Du, Q. Tang, X. Liu, S. Li, J. Du, K. Yan, Y. Li, X. Hao, Y. Shi, L. Pan, *Cell Rep. Phys. Sci.* **2024**, 5, 102025.

[3] a) J.-H. Zhang, Y. Zhang, N. Sun, Y. Li, J. Du, L. Zhu, X. Hao, *Nano Energy* **2021**, 84, 105892; b) H.-W. Park, N. D. Huynh, W. Kim, C. Lee, Y. Nam, S. Lee, K.-B. Chung, D. Choi, *Nano Energy* **2018**, 50, 9.

[4] a) J.-H. Zhang, X. Hao, *Nano Energy* **2020**, 76, 105074; b) Y. Park, Y. E. Shin, J. Park, Y. Lee, M. P. Kim, Y. R. Kim, S. Na, S. K. Ghosh, H. Ko, *ACS Nano* **2020**, 14, 7101.

[5] J. Chen, H. Guo, X. He, G. Liu, Y. Xi, H. Shi, C. Hu, *ACS Appl. Mater. Interfaces* **2016**, 8, 736.

[6] a) J. Yan, Y. G. Jeong, *ACS Appl. Mater. Interfaces* **2016**, 8, 15700; b) W. B. Ko, D. S. Choi, C. H. Lee, J. Y. Yang, G. S. Yoon, J. P. Hong, *Adv. Mater.* **2017**, 29, 1704434.

[7] D.-S. Park, M. Hadad, L. M. Riemer, R. Ignatans, D. Spirito, V. Esposito, V. Tileli, N. Gauquelin, D. Chezganov, D. Jannis, J. Verbeeck, S. Gorfman, N. Pryds, P. Muralt, D. Damjanovic, *Science* **2022**, 375, 653.

[8] J. Y. Fu, W. Zhu, N. Li, N. B. Smith, L. E. Cross, *Appl. Phys. Lett.* **2007**, 91, 182910.

[9] W. Zeng, X.-M. Tao, S. Chen, S. Shang, H. L. W. Chan, S. H. Choy, *Energy Environ. Sci.* **2013**, 6, 2631.
